# Supplementary material for: Replay bursts in humans coincide with activation of the default mode and parietal alpha networks
Source: Neuron. 2021 Mar 3;109(5):882–893.e7. doi: 10.1016/j.neuron.2020.12.007 (PMC7927915; doi:10.1016/j.neuron.2020.12.007)
Supplement: Document S1. Figures S1–S6 [file mmc1.pdf]

Neuron, Volume *109*

Supplemental information

Replay bursts in humans coincide with activation of the default mode and parietal alpha networks

Cameron Higgins, Yunzhe Liu, Diego Vidaurre, Zeb Kurth-Nelson, Ray Dolan, Timothy Behrens, and Mark Woolrich

Supplementary Information

This PDF file includes:

Figs. S1 to S6

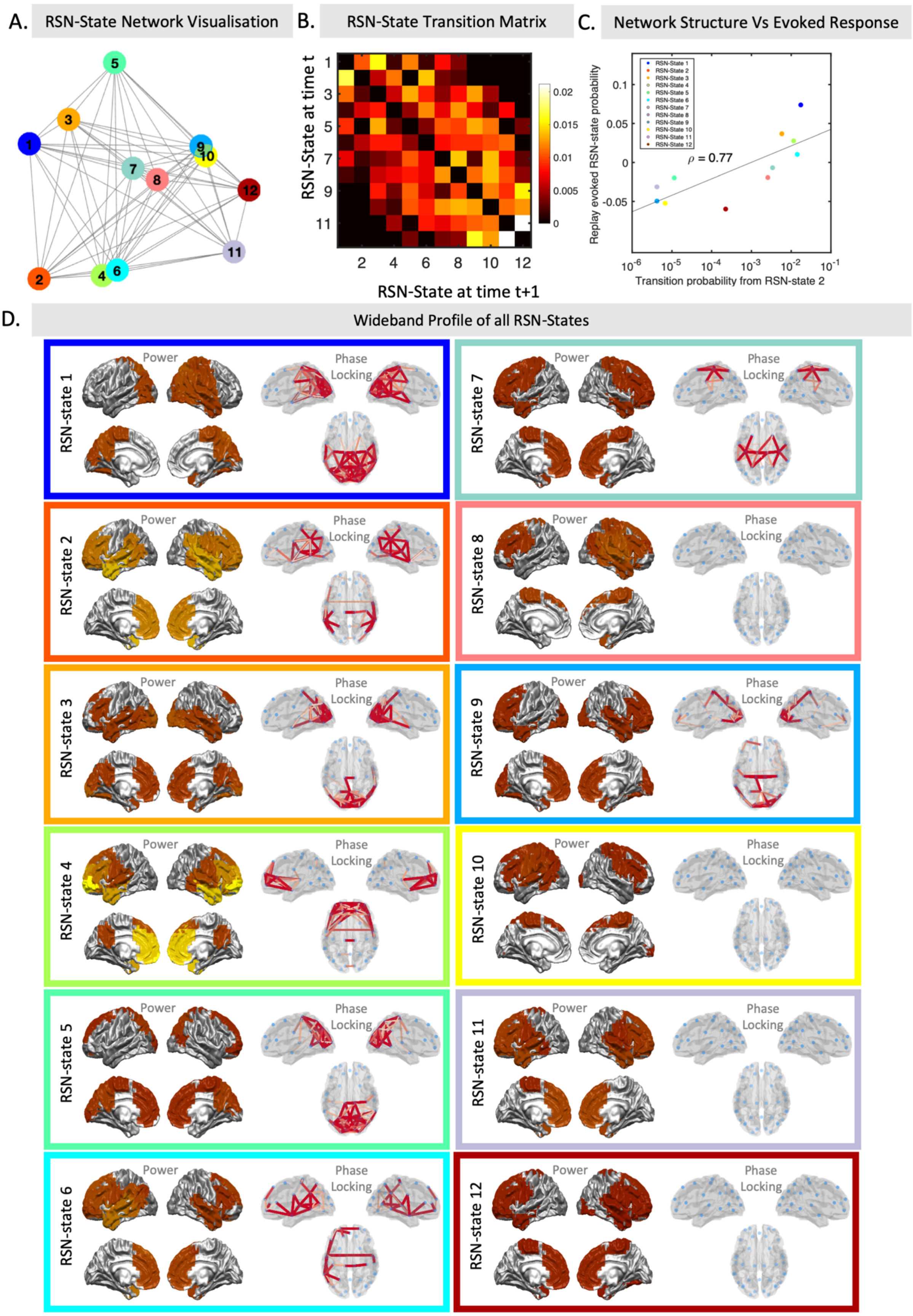
A. RSN-State Network Visualisation
B. RSN-State Transition Matrix
C. Network Structure Vs Evoked Response
RSN-State at time t
RSN-State at time t+1
Replay evoked RSN-state probability
Transition probability from RSN-state 2
ρ = 0.77
D. Wideband Profile of all RSN-States
Power
Phase Locking
RSN-state 1
RSN-state 2
RSN-state 3
RSN-state 4
RSN-state 5
RSN-state 6
RSN-state 7
RSN-state 8
RSN-state 9
RSN-state 10
RSN-state 11
RSN-state 12

Fig. S1.

Resting State Network Profiles; related to Figure 2. (A) The RSN-states can be visualized as a full network through a multidimensional scaling of the transition matrix (see STAR Methods). We label RSN-states 1-12 in order of their appearance on the horizontal axis of this graph, accounting for the greatest portion of distances between RSN-states. (B) The transition matrix (excluding diagonal entries) for the RSN-states; the diagonal structure reflects this labelling structure, with transitions more probable between adjacent pairs of RSN-states than distant pairs. (C) Correlating the second row of the RSN-state transition matrix on the x axis with the replay evoked RSN-state probability (ie the value at t=0 on Fig. 2A) on the y axis identifies a strong relationship (Pearson's rho=0.77, p=5.1e-3). (D) Wideband profiles for all RSN-states, plotting the wideband power (thresholded at 50%) and coherence (thresholded with Gaussian Mixture Model). See STAR Methods for further details and Supplementary Text for clarification of how these relate to equivalent networks in fMRI.

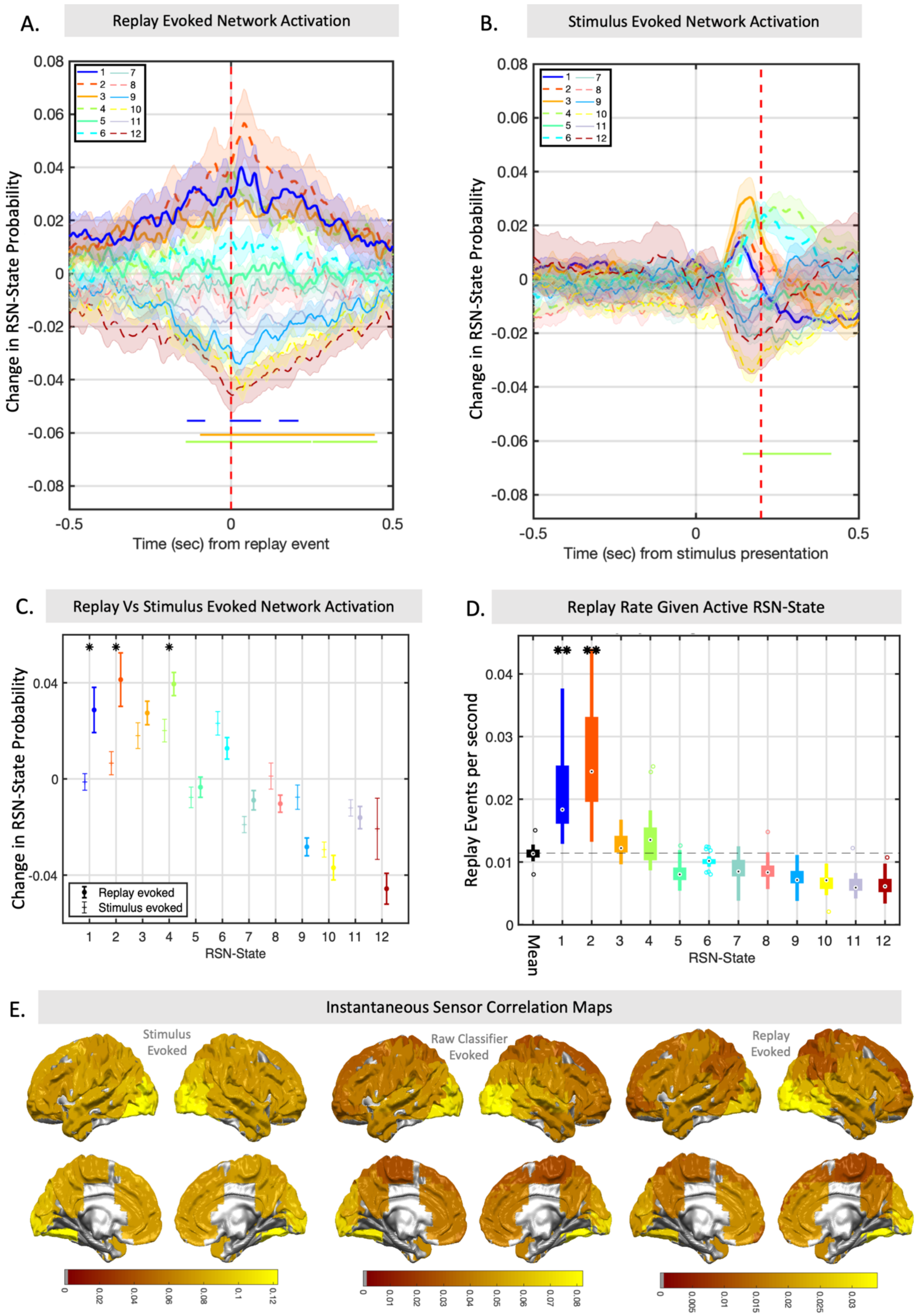

A.
Replay Evoked Network Activation
Change in RSN-State Probability
Time (sec) from replay event
B.
Stimulus Evoked Network Activation
Change in RSN-State Probability
Time (sec) from stimulus presentation
C.
Replay Vs Stimulus Evoked Network Activation
Change in RSN-State Probability
Replay evoked
Stimulus evoked
RSN-State
D.
Replay Rate Given Active RSN-State
Replay Events per second
Mean
RSN-State
E.
Instantaneous Sensor Correlation Maps
Stimulus Evoked
Raw Classifier Evoked
Replay Evoked

Fig. S2.

Replicating the result of Figure 2 on a second dataset and further justification of results. (A) Replication of Fig. 2A on a second dataset; plotting mean +/- ste of the RSN-state probability evoked by replay onset; replay onset coincides with a broad peak in activity in RSN-states 1 and 2, and to a lesser extent RSN-states 3 and 4. Significance bars show clusters where p<1e-4. (B) Replication of Fig. 2C on a second dataset; mean +/- ste of the RSN-state probability associated with the original stimulus data. Significance bars show clusters where p<1e-4. (C) Replication of Fig. 2D on a second dataset; comparing directly the mean +/- ste of the evoked state distribution at replay time and at the classifier training time identifies RSN-states 1 and 2 as significantly increased during spontaneous replay (multiple paired t tests). Single asterisk denotes p<0.05, double asterisk denotes p<1e-3. (D) Replay rate as a function of active RSN-state; in Fig. 2 we showed that replay onset predicts an increase in RSN-states 1 and 2. Reinforcing this finding, here we analyze this relation in reverse. We show that activation of RSN-states 1 and 2 similarly predict an increase in the rate of replay events. Asterisk denotes significance at p=0.05, double asterisk denotes significance at p=1e-3. (E) Instantaneous sensor correlation maps; putting aside the network patterns analyzed in the main text, we can similarly consider the instantaneous sensor magnitudes and how they correlate with (i) the design matrix entries in the original stimulus encoding data (left); (ii) the raw classifier scores in the resting state data (middle); or (iii) the replay scores in the resting state data. This analysis confirms the underlying evidence behind each of these signals are representations predominantly in visual cortical areas.

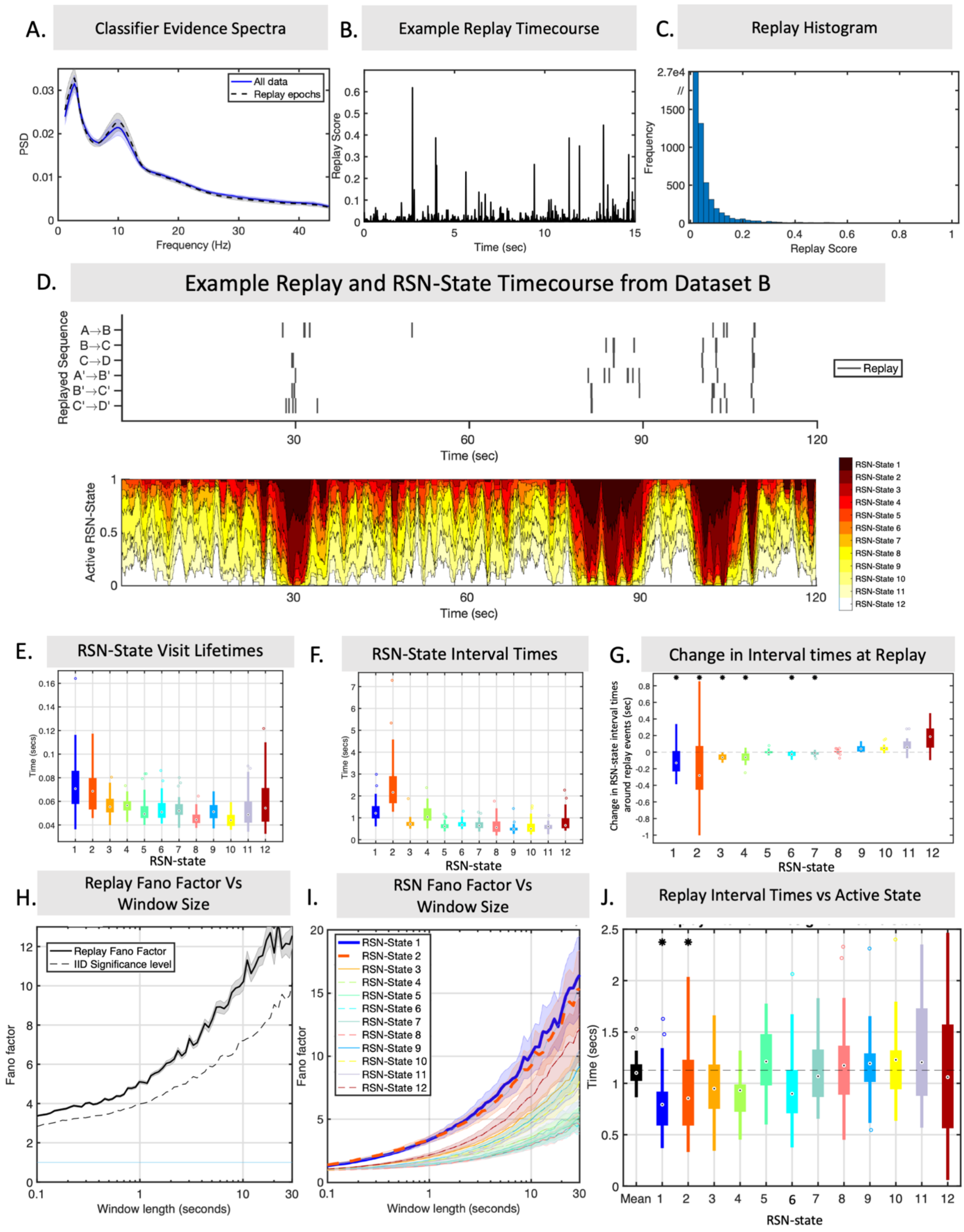

Fig. S3.

Replicating the results of Figure 3 on a second dataset and characterizing the replay timecourse. (A) The classifier evidence spectra reproduces peaks in alpha and delta/theta modes characterized in the text. The two lines plot the power spectrum of the classifier timecourses before the logistic sigmoid transform is applied; this is separated by replay epochs and non-replay epochs as a baseline. This analysis reproduces two prominent peaks in the alpha and delta/theta range broadly characterized in the data, showing that neither of these

features are removed by the classifiers. These peaks are accentuated when we time-lock this analysis to epochs surrounding replay events; extracting the same spectral modes in this data as used in the main manuscript text (see STAR Methods), we find a prominent increase in the alpha range (paired t-test, p=0.002) and a weaker but significant increase in the delta/theta range (paired t-test, p=0.045). (B) Example replay timecourse from one subject. (C) Histogram of replay timecourse values; this timecourse is very sparse, with the vast majority of values very close to zero. This justifies the binarized dynamics analysis pursued in the text over alternative spectral analysis methods. (D). Replication of Fig 3A on second dataset; example data from one subject of the replay event times (upper) and one-second moving average RSN-state probabilities (lower), suggesting both replay and RSN-state visits are not distributed evenly over long timescales. (E) Group level RSN-state visit lifetimes, characterizing the variation in mean lifetime statistics over subjects. (F) Group level RSN-state interval times, characterizing the variation in mean interval time statistics over subjects. (G) Change in interval times at replay; the mean RSN-state interval times at replay (defined as a state occurrence within 250msec of a replay event) minus the mean RSN-state interval times outside replay periods. This identifies a number of RSN-states, most prominently RSN-states 1 and 2, as having shorter interval times around replay events, confirming these state visits cluster together around replay events. Asterisk denotes significance with left-tailed paired t-test at p<0.05. (H) Replication of Fig. 3C on a second dataset; temporal irregularity can be quantified by looking at the Fano factor as a function of window size. Replay events show that this irregularity measure increases over longer timescales, displaying maximum temporal irregularity over windows of ten seconds or more. (I) Replication of Fig. 3D on a second dataset; this structure is replicated by the RSN-state activations, with RSN-states 1 and 2 displaying the most irregular patterns at long timescales. (J) Replication of Fig. 3E; this temporal structure is not just common but in fact coincides; replay events that occur during RSN-state 1 or 2 have significantly shorter intervals, reflecting rapid bursty behavior during the infrequent state visits and long periods of quiescence outside of these. Asterisk denotes significant deviation from mean at p<0.05.

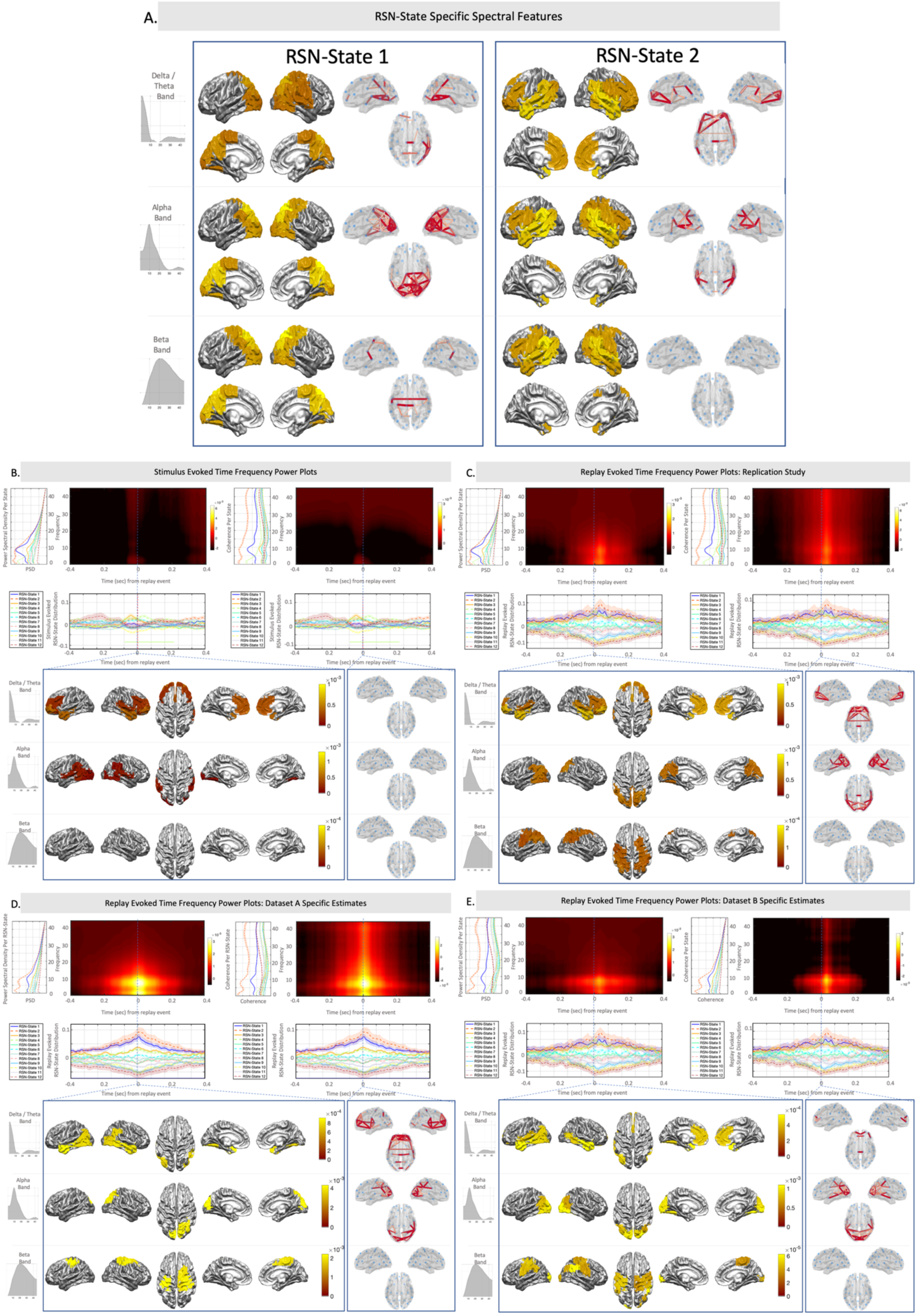
A.
RSN-State Specific Spectral Features
RSN-State 1
RSN-State 2
Delta / Theta Band
Alpha Band
Beta Band
B.
Stimulus Evoked Time Frequency Power Plots
C.
Replay Evoked Time Frequency Power Plots: Replication Study
D.
Replay Evoked Time Frequency Power Plots: Dataset A Specific Estimates
E.
Replay Evoked Time Frequency Power Plots: Dataset B Specific Estimates
Power Spectral Density Per State
Coherence Per State
Frequency
PSD
Coherence
Time (sec) from replay event
Stimulus Evoked RSN-State Distribution
Replay Evoked RSN-State Distribution

Fig. S4.

Replication of Figure 5 results on a second dataset and further spectral profile characterizations. (A) The full spectro-spatial activity maps associated with RSN-states 1 and 2; where Fig. S1 plotted the wideband distribution of power, this can be further analyzed using a data driven spectral decomposition. We here plot the spatial distribution of power in each frequency band (thresholded at 50%); along with the coherence network (Gaussian mixture model threshold; see STAR Methods). This supports our interpretation of RSN-state 1 as the parietal alpha network, given the prominence of the alpha peak over parietal areas; this pattern of electrophysiology activity has been shown in simultaneous EEG/fMRI experiments to anticorrelate with the fMRI Dorsal Attention Network (Mantini et al., 2007; Sitnikova et al., 2020), supporting the interpretation that it is associated with the DAN 'switching off'. These spectral maps further support our interpretation of RSN-state 2 as reflecting the MEG DMN equivalent; further to the broadband patterns of activity in parietal cingulate and lateral parietal areas of Fig S1, the narrowband signal identifies frontal, anterior cingulate and lateral temporal areas active in a lower frequency band to the dominant alpha band. This is consistent with the previous MEG literature on the DMN (Vidaurre et al., 2018) and with simultaneous EEG and fMRI recordings of the EEG correlates of the fMRI DMN network (Mantini et al., 2007; Sitnikova et al., 2020). (B) As a control, we can apply the same analysis of Fig. 5 to the original stimulus encoding data. Using the original stimulus-evoked RSN-state probabilities as weights for the spectral information unique to each RSN-state allows a reconstructed time-frequency estimate of power spectral density and coherence around stimulus presentation. This does not replicate the same level of power and coherence seen around replay events, confirming these patterns are unique to spontaneous replay. (C) Replication of Fig. 5 using a second dataset; using the replay-evoked RSN-state probabilities as weights for the spectral information unique to each RSN-state allows a reconstructed time-frequency estimate of power spectral density and coherence around replay events, revealing a prominent peak in the alpha and delta /theta bands. Plotting the spatial distribution of activity in the defined frequency modes at the time of replay identifies independent modes of coherent activity; a low frequency mode (top panel) comprising frontal DMN and temporal areas, and an alpha frequency mode (middle panel) comprising parietal DMN regions and visual cortex. (D) Replication of Fig. 5 using Dataset A spectral profiles; Fig. 3 was generated using RSN-state spectral information learned from Dataset C (see STAR Methods). The same plot can be reproduced using the spectral information from Dataset A itself, reproducing the same features of the main plot. (E) Replication of Fig. S4C using Dataset B spectral profiles; similarly Fig S4C was generated using RSN-state spectral information learned from Dataset C, but can be replicated using spectral information learned from Dataset B. For ease of visualization and comparison of the full spatial patterns, power spectra in B-E were thresholded at 75%; coherence networks were thresholded with a Gaussian mixture model (see STAR methods).

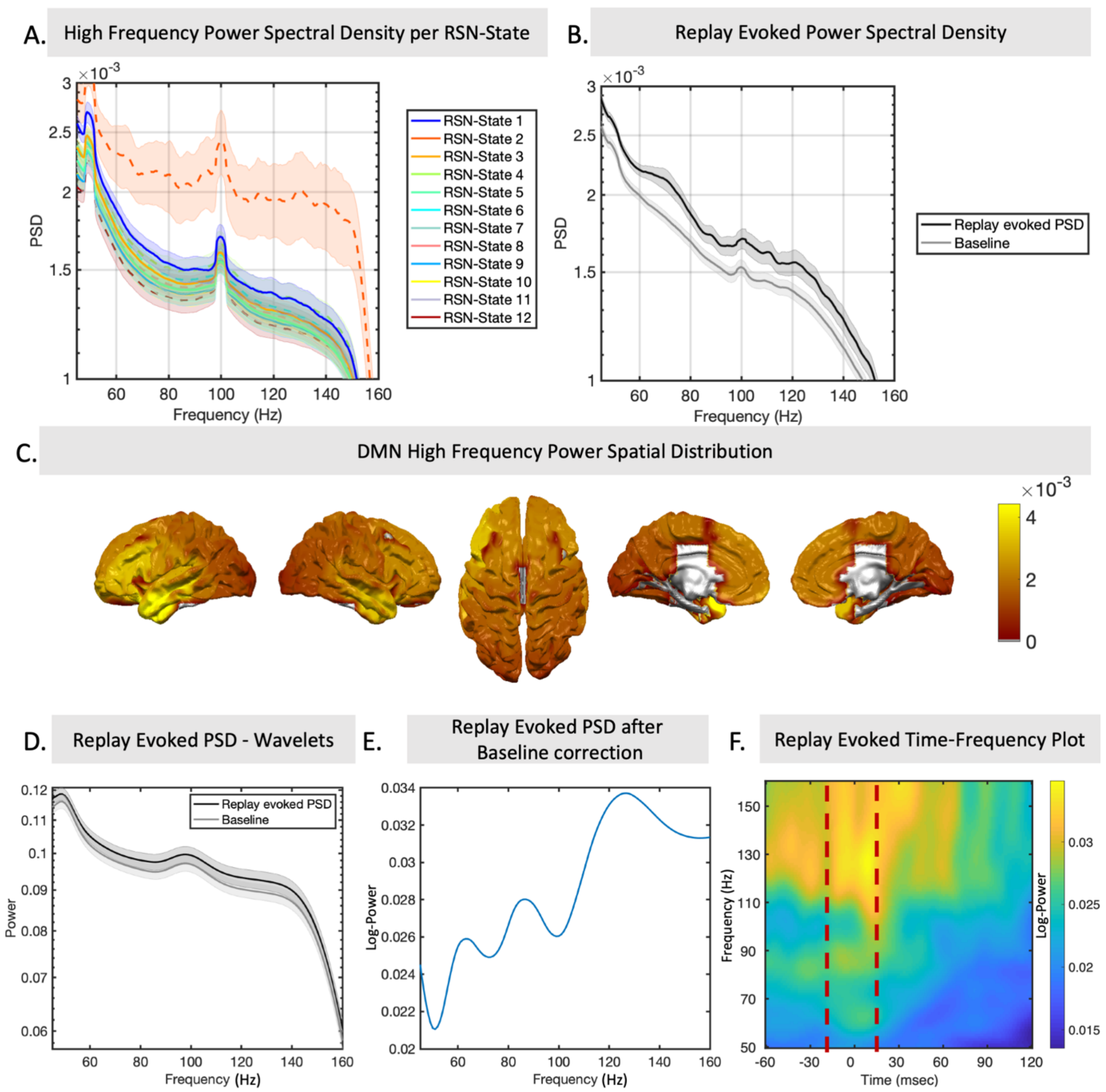

Fig. S5.

Replication of the result of Figure 6 on a second dataset. (A) Although the RSN-state model was originally fit to data filtered at 1-45Hz, we can still analyze whether the state timings correlate with specific patterns in frequency bands outside this range in the original data. This reveals a very strong association between the DMN state and power in high frequencies – despite these high frequencies not having been originally included in the model. (B) Similarly, the onset of replay is associated with an increase in high frequency power relative to the global average. Replay-evoked power computed by taking 50msec windows of data around identified replay events; for baseline see STAR Methods. (C) The spatial distribution of high frequency (102-148Hz) power in the DMN state; activity in this RSN-state and in this frequency band source localizes to temporal cortex. (D) To compare our findings directly with previously reported results (Liu et. al. 2019), we recreated Fig. 6B using a wavelet decomposition applied to the entire timeseries (see STAR Methods). Taking the same 30msec window around replay events replicates the broadband high frequency spectrum of Fig. 6B. (E) The replay evoked PSD after baseline correction; that is, the mean difference between a subject's replay-evoked log power and their log baseline power. This produces a stronger peak in higher frequencies than is apparent in D. (F) Plotting the full

time-frequency response at replay epochs after baseline correction reproduces the result reported by Liu et. al. 2019 that appears more narrowband than is apparent in D. Dotted red lines define the window that is used for 'replay evoked power' in D and E. A number of small differences remain in our preprocessing pipelines – specifically the use of beamformed and parcellated data as opposed to raw sensor signals, and the use of a 99% threshold as opposed to a 95% threshold when defining replay events – but the result reported by Liu et. al. 2019 is replicated here independently of those differences. Thus, we conclude that the relatively broadband appearance of the signal shown in Fig 6B appears more narrowband following baseline correction, which explains the apparent difference between this figure in our paper and the results previously reported by Liu et. al. 2019.

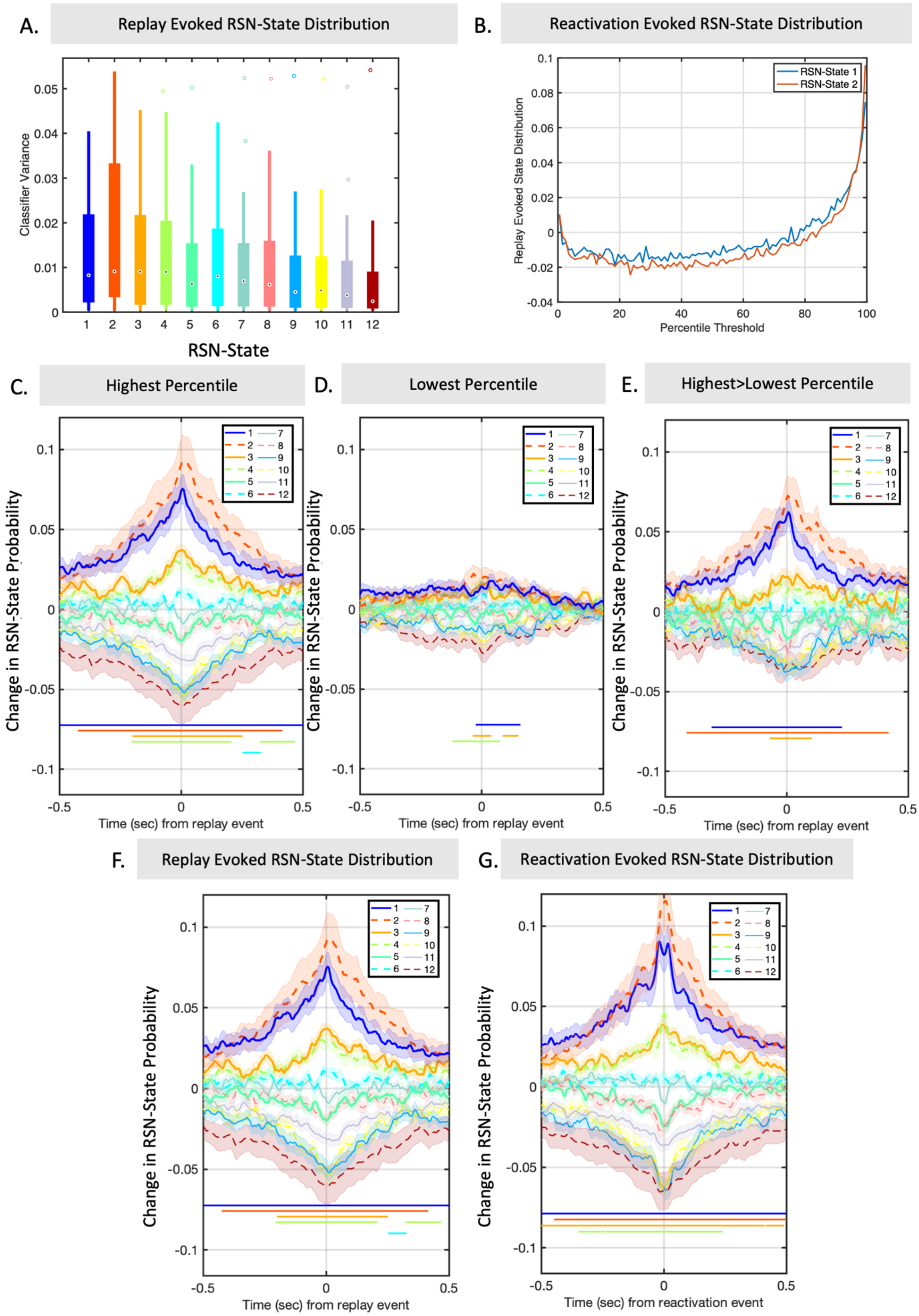
A.
Replay Evoked RSN-State Distribution
Classifier Variance
RSN-State
B.
Reactivation Evoked RSN-State Distribution
RSN-State 1
RSN-State 2
Replay Evoked State Distribution
Percentile Threshold
C.
Highest Percentile
D.
Lowest Percentile
E.
Highest>Lowest Percentile
Change in RSN-State Probability
Time (sec) from replay event
F.
Replay Evoked RSN-State Distribution
G.
Reactivation Evoked RSN-State Distribution
Time (sec) from reactivation event

Fig. S6.

Further controls and analyses; related to STAR Methods and Figure 2. (A) Variance of classifier output varies as a function of RSN-state (One way ANOVA, $p < 0.01$). Box plots show variation over subjects of observed classifier variance during activation of each RSN-state. (B) Focusing only on RSN-states 1 and 2, the replay evoked state distribution at $t=0$ (ie the exact time of estimated replay onset) is plotted against the percentile threshold used. This shows that the association with RSN-states 1 and 2 reported in Fig. 2A is robust over a broad range of percentile thresholds. It similarly performs the variance control outlined in the STAR Methods; this identifies a small effect emerging for the lowest 1 percentile, suggesting that the classifier variance does in some way contribute to the RSN-state replay relationship, however the effect is much weaker than the main result. (C) Replicating the main result of Fig. 2A, the RSN-state distribution evoked by the highest percentile of replay scores, for comparison. (D) The RSN-state distribution evoked by the lowest percentile of replay scores. This effect however is not significant after Bonferroni correction, and is only a fraction of the effect size of (D). (E) Taking the contrast of these as a control for the effect size that is solely due to classifier variance again replicates a strong association between RSN-states 1 and 2 and the onset of replay, confirming our interpretation of the main result. (F) The replay evoked RSN-state distribution is a special case of the reactivation evoked RSN-state distribution; the main result of Fig. 2A, reproduced for ease of comparison, with (G) the corresponding RSN-state distribution evoked by reactivation (see STAR Methods and Supplementary Text). Significance bars denote clusters with $p<1e-4$. We find no evidence of distinct relationships between RSN-states and reactivation as opposed to replay, however also caution against any strong interpretation of this result given the fundamental limitations of our methods to make such a direct comparison (see STAR methods).
