# Supplementary material for: Replay bursts in humans coincide with activation of the default mode and parietal alpha networks
Source: Neuron. 2021 Mar 3;109(5):882–893.e7. doi: 10.1016/j.neuron.2020.12.007 (PMC7927915; doi:10.1016/j.neuron.2020.12.007)
Supplement: Document S2. Article plus supplemental information [file mmc2.pdf]

Neuron

Article

Replay bursts in humans coincide with activation of the default mode and parietal alpha networks

Highlights

- Replay in humans coincides with activity in specific resting brain networks
- Clusters of heightened default mode and alpha activity are linked to replay bursts
- These networks are characterized by highly synchronized brain-wide oscillations
- High-frequency power bursts are uniquely linked to default mode network activation

Authors

Cameron Higgins, Yunzhe Liu, Diego Vidaurre, Zeb Kurth-Nelson, Ray Dolan, Timothy Behrens, Mark Woolrich

Correspondence

cameron.higgins@ohba.ox.ac.uk

In Brief

Our brains form stable memory traces by spontaneously replaying recently acquired information. Higgins et al. show that this process coincides with activity in specific resting-state brain networks, with bursts of replay events occurring selectively during clusters of heightened default mode and parietal alpha network activity.

Higgins et al., 2021, Neuron *109*, 882–893
March 3, 2021 © 2020 The Authors. Published by Elsevier Inc.
https://doi.org/10.1016/j.neuron.2020.12.007

Neuron

Article

Replay bursts in humans coincide with activation of the default mode and parietal alpha networks

Cameron Higgins,[1,6,8,*] Yunzhe Liu,[2,3,6] Diego Vidaurre,[1,4] Zeb Kurth-Nelson,[3,5] Ray Dolan,[2,3] Timothy Behrens,[1,2,7] and Mark Woolrich[1,7]
[1]Wellcome Centre for Integrative Neuroimaging, University of Oxford, Oxford, UK
[2]Wellcome Trust Centre for Neuroimaging, University College London, London, UK
[3]Max Planck University College London Centre for Computational Psychiatry and Ageing Research, University College London, London, UK
[4]Center of Functionally Integrative Neuroscience, Department of Clinical Medicine, Aarhus University, Aarhus, Denmark
[5]Deepmind, London, UK
[6]These authors contributed equally
[7]Senior author
[8]Lead contact
*Correspondence: cameron.higgins@ohba.ox.ac.uk
https://doi.org/10.1016/j.neuron.2020.12.007

Summary

Our brains at rest spontaneously replay recently acquired information, but how this process is orchestrated to avoid interference with ongoing cognition is an open question. Here we investigated whether replay coincided with spontaneous patterns of whole-brain activity. We found, in two separate datasets, that replay sequences were packaged into transient bursts occurring selectively during activation of the default mode network (DMN) and parietal alpha networks. These networks are believed to support inwardly oriented attention and inhibit bottom-up sensory processing and were characterized by widespread synchronized oscillations coupled to increases in high frequency power, mechanisms thought to coordinate information flow between disparate cortical areas. Our data reveal a tight correspondence between two widely studied phenomena in neural physiology and suggest that the DMN may coordinate replay bursts in a manner that minimizes interference with ongoing cognition.

Introduction

A key mechanism by which the brain forms and stores new knowledge is neural replay, where the patterns of neural activity associated with specific items are spontaneously reinstated in structured sweeps (Wilson and McNaughton, 1994). These sweeps project to widespread regions of the cortex (Ji and Wilson, 2007; Qin et al., 1997), with physiological signatures known as sharp wave ripples that have been described as the most synchronous events in the mammalian brain (Buzsáki, 2015). Such spatially dispersed patterns of activity are often initiated during specific states, such as slow-wave sleep, presumably to preclude interference with ongoing wakeful processes. Replay was originally discovered during sleep but is now known to also occur during wakefulness, particularly within periods of immobility and rest (Carr et al., 2011; Foster, 2017; Kudrimoti et al., 1999). An unanswered question in neuroscience is how the brain orchestrates these structured events in a manner that minimizes interference with ongoing cognition.

Neuroimaging studies have long highlighted that, during wakeful rest, the brain displays an intrinsic activity structure, cycling through a series of canonical resting-state networks (RSNs) (Biswal et al., 1995; Brookes et al., 2011; Fox and Raichle, 2007). One network that has drawn particular attention is the default mode network (DMN), a disparate set of brain regions that coactivate during "offline" periods, such as between trials in the absence of specific tasks as well as during sleep (Fukunaga et al., 2006; Raichle et al., 2001). The DMN has since been identified as correlating with a number of introspective cognitive states, such as episodic memory and future-oriented thought, suggesting a functional role during wakeful rest in mediating internally generated cognition and inhibiting bottom-up sensory processing (de Pasquale et al., 2010). Magnetoencephalography (MEG) resting-state studies have likewise detected DMN activation (along with that of many other networks) with millisecond temporal resolution, demonstrating that these networks activate transiently within specific spectrally defined modes (Baker et al., 2014; Brookes et al., 2011; de Pasquale et al., 2010; Vidaurre et al., 2018). An ability to measure spontaneous replay noninvasively in humans has now been demonstrated (Kurth-Nelson et al., 2016; Liu et al., 2019, 2020), and this allowed us to investigate a potential link between replay and resting brain network activity.

Replay during slow-wave sleep is associated with specific electrophysiological patterns; low-frequency oscillations synchronize widespread regions of the cortex, whereas high-frequency sharp wave ripples propagate between the hippocampus and cortical areas (Buzsáki, 2015; Chrobak and

 © 2020 The Authors. Published by Elsevier Inc.
This is an open access article under the CC BY license (http://creativecommons.org/licenses/by/4.0/).

CellPress
OPEN ACCESS

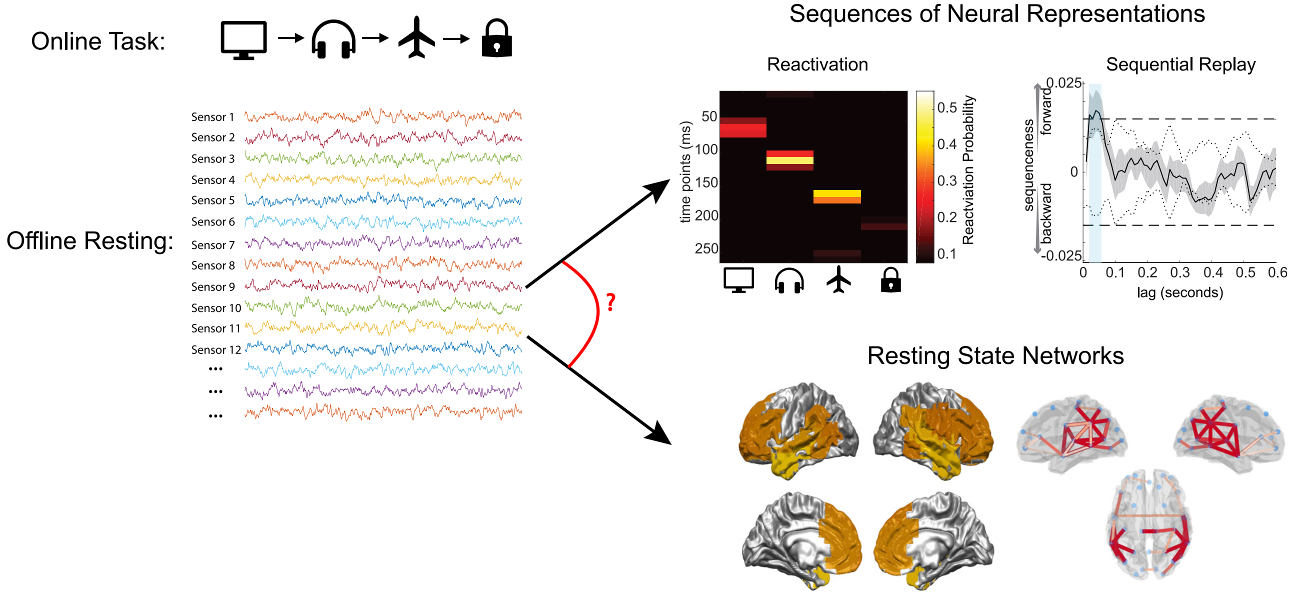

Figure 1. Task outline

The online task required participants to learn and remember a sequence of items. During the offline resting period, subjects were recorded passively with no immediate task; they were later tested for correct recall of the item sequence. The data from the offline resting period were analyzed in two ways. The first analysis detected replay using the methods of Liu et al. (2019), using classifiers trained on the task items and identifying periods when they are reactivated in the specific sequence required by the task. The second analysis identified when specific RSNs were activated by using an established model (Vidaurre et al., 2018) to detect spontaneous patterns of spatial and spectral activity in the data. The objective was to determine whether the two measures were linked.

Buzsáki, 1996). These widespread patterns appear to be integral for effective function of replay in consolidating memories (Buzsáki, 1998). In contrast, the wakeful brain displays a markedly different profile, with transient periods of synchronous activity interspersed with widespread desynchronization associated with distributed processing of parallel cognitive tasks (Brookes et al., 2011; Vidaurre et al., 2018). Here we wanted to find out whether replay events as detected by Liu et al. (2019) are linked to specific changes in whole-brain neural activity, changes that might explain how wakeful replay could reinstate distributed cortical patterns from memory without interference from competing cognitive demands.

Results

Spontaneous replay coincides with activation of the default mode and parietal alpha networks

We investigated whether the human replay events discovered by Liu et al. (2019), each representing rapid serial reactivation of learned stimulus representations (Figure 1), coincided with specific macro patterns of resting-brain network activity studied widely in the literature (Baker et al., 2014; Brookes et al., 2011; de Pasquale et al., 2010; Vidaurre et al., 2018). The focus of our analysis was the same MEG scan data of Liu et al. (2019), collected during resting periods of their experiment.

We first repeated the analysis of Liu et al. (2019) to identify specific moments when replay occurred within these resting-state data. Briefly, we trained multivariate classifiers to recognize each experimental stimulus, applied these classifiers to the resting data, and found that the times when classifiers detected stimulus representations played out in rapid sequences in an order defined by the task (STAR methods; Liu et al., 2020).

Next, in the same data, we determined which of a set of canonical RSN states were active at each point in time (STAR methods; Figure 1). We inferred 12 RSN states in a data-driven way using an established hidden Markov model framework (STAR methods; Vidaurre et al., 2018). These RSN states were labeled according to multidimensional scaling of their distances from each other (STAR methods; Figure S1); thus, RSN states 1 and 12 represented opposing extremes of a single major axis of differentiation between networks. We then conducted an evoked response analysis to find out whether activation of the RSN states was modulated around replay events.

As shown in Figure 2A, a strong relationship emerged between replay onset and two RSN states in particular: RSN states 1 and 2. This relationship peaked at around $t = 0$, the exact time of replay onset, but exhibited a decay at either side of this time, with a statistically significant association up to 0.5 s before and after each estimated replay onset time (non-parametric cluster significance test, $p < 2e{-}4$ for both RSN states). In addition, a weaker relationship was also evident between RSN states 3 and 4 and the observed replay times ($p < 2e{-}4$ for both RSN states). The remaining RSN states display a pattern of decreasing activation that appears approximately in ordinal sequence. Given that the RSN state labels were based on the transition matrix (STAR methods; Figure S1), we then correlated the strength of each RSN state's replay association with its probability of activation immediately following RSN state 2 (the state most strongly

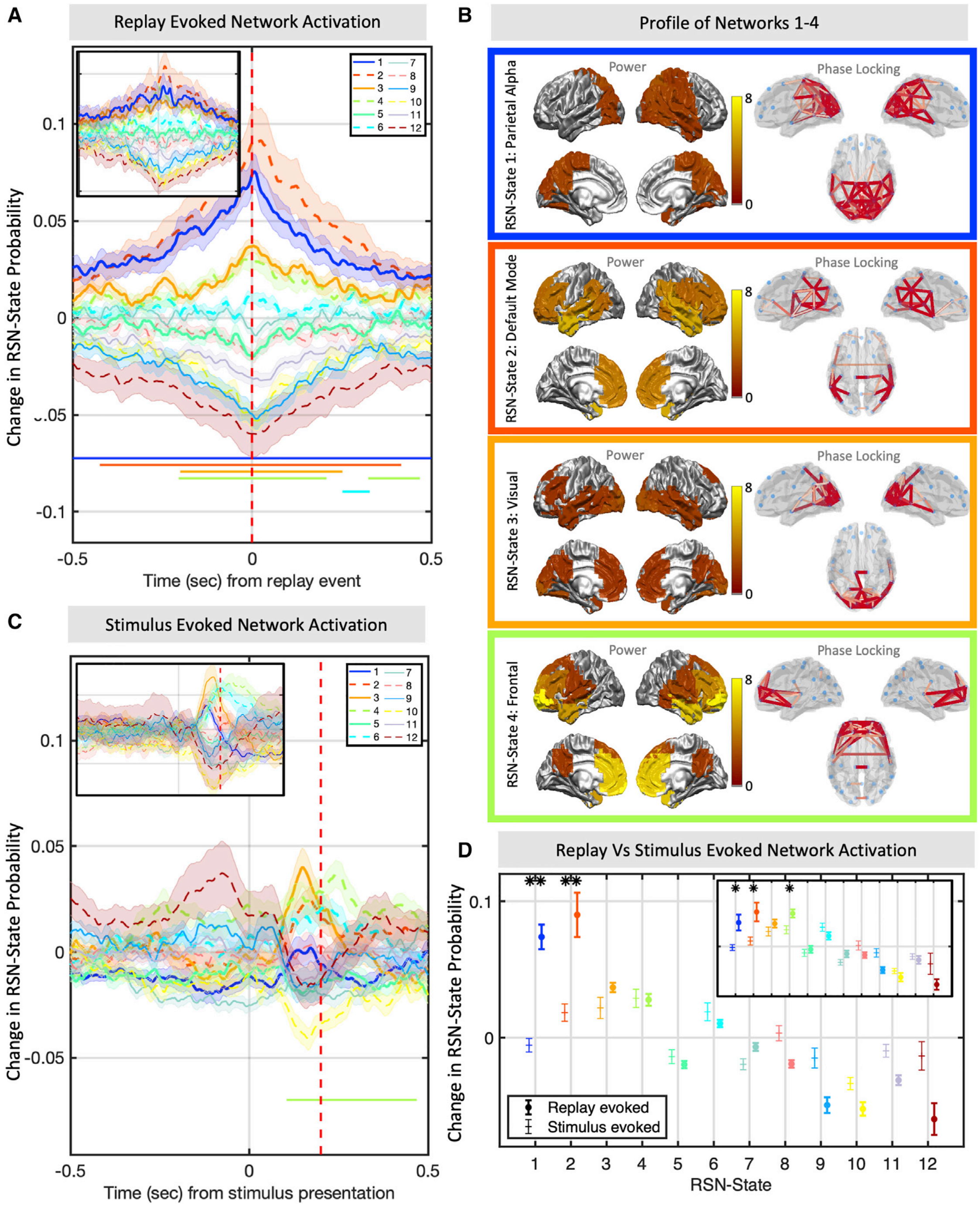

Figure 2. Replay coincides with activation of specific RSNs

(A) Mean ± SEM of the change in RSN state probability around replay events. A strong correlation exists between the replay times identified by Liu et al. (2019) and resting network activity; namely, RSN states 1 and 2. This association peaks at t = 0, representing the exact time of replay onset, but remains significant up to 0.5 s to either side of each event, showing that activity in either of these RSN states is broadly predictive of replay. Significance bars show clusters where $p < 1e{-3}$. Inset: result of the replication study on the second dataset.

(B) The broadband power and coherence networks that characterize each state identify RSN states 1 and 2 as the parietal alpha network and DMN, respectively. RSN states 3 and 4 correspond to activity in the visual and frontal areas, respectively. Power maps were thresholded at 50%, and phase-locking networks were thresholded with a Gaussian mixture model (STAR methods).

(legend continued on next page)

associated with replay) and found a very strong relationship (Pearson's Rho = 0.77, p = 5.1e−3). Thus, the probability of any RSN state activating at the time of replay is determined by that state's probability of proceeding fromRSN state 2.

Each RSN state can be described by its distinct spectral power and phase-locking profile; these profiles are summarized by averaging over frequency bands in Figure 2B for RSN states 1–4. This highlights that RSN state 1 was associated with activity over the parietal cortex; the equivalent network in simultaneous electroencephalogram (EEG)-fMRI studies has been shown to be anticorrelated with the dorsal attention network (DAN) (Mantini et al., 2007; Sitnikova et al., 2020); thus, activation of RSN state 1 corresponds to the DAN switching off. RSN state 2 combines high-power signals in frontal and temporal regions with coherent oscillations in the lateral parietal cortex, regions that comprise the DMN. RSN state 3 can be interpreted as activation of the visual cortex, and RSN state 4 can be interpreted as activation of frontal cortical regions. The profiles of the remaining RSN states are shown in Figure S1.

The alignment of replay with specific RSN states would not be surprising if some of these networks simply reflected the patterns of activity present during the original stimulus encoding in the functional localizer data. Because our methods are based on linear classifiers, we expect the instantaneous patterns between stimulus encoding and replay to reflect activity in roughly the same visual areas (and show that this is indeed the case in Figure S2E); but it is not clear whether whole-brain network patterns of activity would be the same. Figure 2C shows that they are not; if we take the same canonical RSN states, hold them fixed, and fit them to the original functional localizer data on which the classifiers were trained, then we identify a markedly different relationship with the RSN states compared with replay. No significant increase was observed to RSN state 1 (p = 0.88 at t = 0.2 s), and RSN state 2 showed a mild increase that was not significant after Bonferroni correction (p = 0.006 at t = 0.2 s). Directly comparing the distribution of RSN states evoked by replay and by stimulus identified a clear differential distribution with highly significant increases in RSN states 1 and 2 (Figure 2D; one-sided t test, p = 8.9e–7 for RSN state 1 and p = 1.9e−4 for RSN state 2). Although RSN states 3 and 4 do show a significant association with replay, this is only in the sense that they replicate patterns of activity associated with the original stimulus-encoding data. Thus, we conclude that brain-wide patterns of resting RSN state activity that are associated with replay encompass both common and unique patterns of activity with respect to the original stimulus-encoding data. Patterns unique to spontaneous replay are better characterized by activation of the default mode and parietal alpha RSN states.

To ensure replicability of these results, we conducted the same analysis in a second independent study that examined replay data in MEG using a very similar but slightly amended paradigm (Liu et al., 2019). We replicated the exact findings, showing that RSN states 1 and 2 had a strong association with replay (Figure 2A, inset; Figure S2; p < 2e−4 for RSN states 1–4; inflexible direct cluster comparison, p < 0.05 for RSN state 1 and RSN state 2). Neither RSN state 1 nor RSN state 2 displayed a significant increase in relation to the functional localizer data (p = 0.67 for RSN state 1 and p = 0.14 for RSN state 2). A paired t test also confirmed that these RSN states were more strongly associated with replay than with the original training data (p = 5.4e−3 for RSN state 1 and p = 9.1e−3 for RSN state 2).

Transient replay bursts coincide with clusters of DMN and parietal alpha network activity

The correlation between replay and the RSN states shown in Figure 2B was maintained for over 0.5 s either side of each replay event. This is difficult to immediately reconcile with the highly transient nature of MEG RSN states, which typically activate for less than 100 ms but also display dispersive long-term temporal statistics (Figure 3B). Replicating previous findings (Baker et al., 2014; Vidaurre et al., 2018), we found that RSN states 1 and 2 displayed longer average lifetimes than any other RSN states and also quiesced for longer periods than any other RSN states (Figure 3B). A qualitative assessment suggested that neither replay nor RSN state activations were distributed evenly over long timescales, with clusters of heightened DMN and parietal alpha network activity in particular coinciding with bursts of replay events (Figure 3A).

To test whether replay events were concentrated into transient bursts, as suggested by Liu et al. (2019), we first computed the Fano factor over the time course of replay events; Fano factors equal to 1 correspond to a regular, non-bursting process, whereas Fano factors greater than 1 correspond to increasingly irregular bursting. As shown in Figure 3D, the observed replay Fano factor increased as a function of window size and exceeded 1 for all window sizes tested, showing that the occurrence of replay events was increasingly irregular over longer time periods. The bursting nature of replay events was further supported by rejection of a broader null hypothesis that intervals between replay events were independent and identically distributed (p < 1e−3; STAR methods). For further characterization of the time course of replay and spectral properties of the classifier outputs, see Figures S3A–S3C.

We conducted the same analysis on the time courses of RSN state occurrences (Figure 3E). As with replay events, this showed that visits to different RSN states were also increasingly irregular over longer time periods but displayed a degree of irregularity that was not uniform over the different RSN states (one-way ANOVA, p < 2e−8 for all window sizes tested; this was significant after multiple comparison correction for RSN states and number of windows). This was particularly pronounced for RSN states 1 and 2 (two-sample t test, p < 6.4e−9 for RSN state 1 and p < 0.01 for RSN state 2), the DMN and parietal alpha RSN states that were most strongly correlated with replay. Thus, the RSN states that were most inclined to cluster together into periods of

(C) Fitting the same fixed RSN states to the original stimulus data on which the replay classifiers were trained identifies an overall network profile markedly different from that of the spontaneous replay events. Significance bars show clusters where p < 1e−3. Inset: result of the replication study on the second dataset.
(D) Directly comparing the mean ± SEM of the evoked state distribution at replay time and at the classifier training time identifies RSN states 1 and 2 as significantly increased during spontaneous replay (multiple paired t tests). *p < 0.05, **p < 1e−3. Inset: result of the replication study on the second dataset.

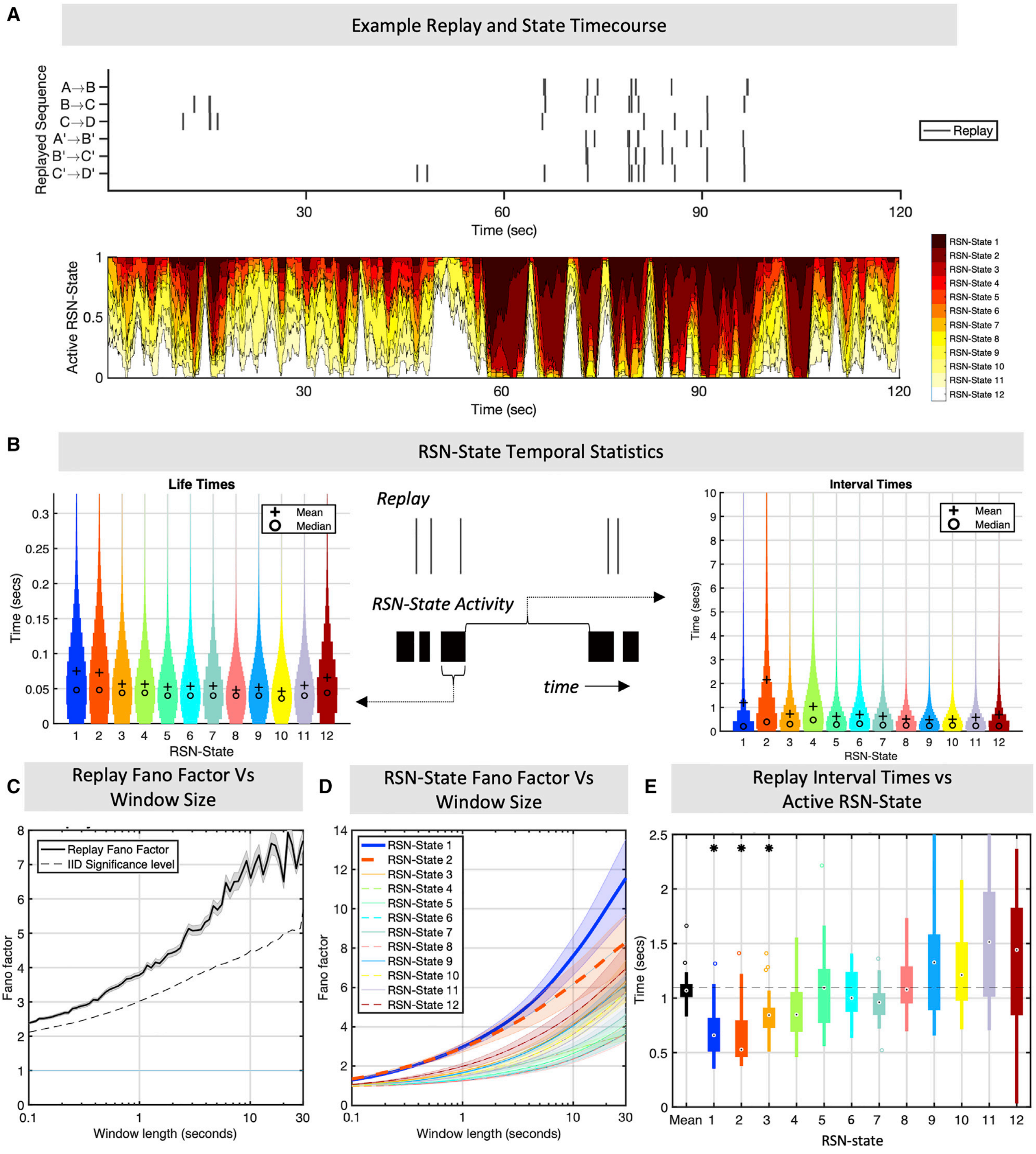

Figure 3. Replay and RSNs share a common long-term temporal structure

(A) Example data from one subject of the replay event times (top) and 1-s moving average RSN state probabilities (bottom), suggesting that replay and RSN state visits are not distributed evenly over long timescales.

(B) RSN state visit temporal statistics and distribution plots showing the RSN state visit lifetimes (left) and interval times (right) along with a schematic showing how these are derived from multiple RSN state visits that may cluster around replay events (center). RSN states 1 and 2 have the longest interval times between successive RSN state visits.

(C) Temporal irregularity can be quantified by looking at the Fano factor (mean over subjects ± SEM) as a function of window size. Replay events show that this irregularity measure increases over longer timescales, displaying maximum temporal irregularity over windows of 10 s or more. Dotted line indicates $p < 1e-3$ permutation threshold for intervals being independent and identically distributed (STAR Methods).

(legend continued on next page)

CellPress
OPEN ACCESS

increased intensity over long timescales were similarly the most correlated with replay.

We have shown evidence that the replay events and RSN state visits display bursty behavior, resulting in clusters of intense activity interspersed with long periods of quiescence. In addition, we have evidence that replay events and RSN state occurrence temporally coincide (cf. Figure 2A). However, this alone does not necessarily mean that the bursting itself temporally coincides. To test whether this was the case, we computed the inter-replay interval time conditioned upon the active RSN state at that time. We found that the interval to the next replay event was significantly determined by the currently active RSN state (one-way ANOVA, $p = 2e{-}9$) so that, when RSN states 1 and 2 were active, there were shorter intervals between replay events ($p = 2.6e{-}3$ and $p = 5e{-}4$, respectively). This suggests that replay events are packaged into bursts that occur selectively during clusters of intense DMN and parietal alpha RSN state activity (for further evidence of RSN state clustering around replay events, see Figure S3G).

To assess reproducibility, we again replicated all results reported here on a second dataset of 22 subjects (Liu et al., 2019; STAR methods). As shown in Figure S3, replay Fano factors again exceeded 1, increased with window size, and exceeded non-parametric permutation test thresholds ($p < 1e{-}3$). Visits to RSN states displayed a similar bursty profile, the degree of which was RSN state dependent ($p < 1.5e{-}8$, one-way ANOVA) with the parietal alpha and DMN RSN states displaying the highest amount (two-sample t test, $p < 7e{-}6$ for RSN state 1, $p < 1.1e{-}4$ for RSN state 2). Inter-replay intervals were again significantly determined by active RSN state ($p = 0.01$, one-way ANOVA). RSN state 1 was significantly associated with shorter intervals ($p = 6e{-}3$), and RSN state 2 was trending in the same direction but not statistically significant ($p = 0.07$).

Replay coincides with distinct patterns of brain-wide highly synchronous activity

Having established a strong temporal association between replay and specific RSN state activation, we next sought to characterize the nature of the brain-wide activity in the replay-associated RSN states. For each RSN state, we calculated the spatial patterns of oscillatory power over all brain regions and the degree of synchronization (coherence) between all brain regions (according to Vidaurre et al., 2018; STAR methods). Figure 4A plots a wide-band summary measure of power and coherence per region of interest (ROI) and RSN state, showing that the replay-associated RSN states (RSN states 1 and 2 and, to a lesser extent, RSN states 3 and 4) were associated with widespread increases in the power and coherence of oscillatory activity compared with the other RSN states (one way ANOVA for group-wise variation, $p < 1e{-}50$ for power and coherence; two-sample t tests, $p < 1e{-}50$ for RSN states 1–4 for power and coherence).

This wide-band summary measure aggregates over frequencies and may therefore be comprised of multiple narrow-band modes in different spatial regions. To better characterize the spatial distribution of activity in distinct frequency bands, we decomposed the spectral activity patterns using non-negative matrix factorization (Vidaurre et al., 2018). This identified three prominent frequency modes reflecting activity in canonical frequency bands of delta/theta, alpha, and beta bands, respectively (Figure 4B). RSN state 2, the DMN, showed a prominent elevation in network coherence across all three frequency bands compared with all other RSN states, whereas RSN state 1, the parietal alpha network, showed increased coherence in the alpha band compared with the other frequency bands. The two other replay-associated RSN states were associated with activity concentrated in the alpha band (RSN state 3) and delta/theta band (RSN state 4).

We also used the RSN state description of the resting state data to calculate the brain-wide patterns of oscillatory power and synchronization occurring specifically around replay events. Figure 5 shows this first as time-frequency plots of power (Figure 5A) and coherence (Figure 5B) averaged over all brain regions, again highlighting a strong increase in power and coherence associated with each replay event. Figure 5C shows the brain-wide patterns of oscillatory power (left) and synchronization (right) that occurred at the time of onset of the replay events. This highlights activity across the frontal and parietal regions of the DMN, with activity in each brain region dissociated into two distinct frequency bands. Frontal nodes of the DMN, including the medial prefrontal cortex and temporal poles, were associated with coherent oscillations in a low delta/theta frequency band; parietal nodes, taken to include the posterior cingulate and lateral parietal cortex, were associated with coherent oscillations in the alpha band.

Again, in the interests of replicability, all of these results were replicated on a second dataset of 22 subjects (Liu et al., 2019; STAR methods), where all activation maps and spectro-spatial profiles were remarkably consistent (Figure S4).

High-frequency power bursts linked exclusively to activation of the DMN

Liu et al. (2019) found an association between the onset of replay and increases in high frequency (>100 Hz) power, consistent with a model of sharp wave ripple activity that coincides with the detected replay events. Given the strong association between replay events and activity in DMN and parietal alpha networks in our work, we wanted to find out whether power in higher-frequency bands might correlate more generally with activity in these networks. Crucially, our approach for estimating the RSN states applied a low-pass filter to the data with a 45-Hz cutoff frequency; thus, any correlation with power spectra above this cutoff can be interpreted as entirely independent of the original RSN state estimation.

(D) This structure is replicated by the RSN state activations, with RSN states 1 and 2 (mean Fano factor over subjects ± SEM) displaying the most irregular patterns at long timescales.

(E) This temporal structure is not just common but coincides; replay events that occur during RSN state 1 or 2 have significantly shorter intervals, reflecting rapid bursty behavior during the infrequent state visits and long periods of quiescence outside of these. Significant deviation from the mean at *$p < 0.05$.

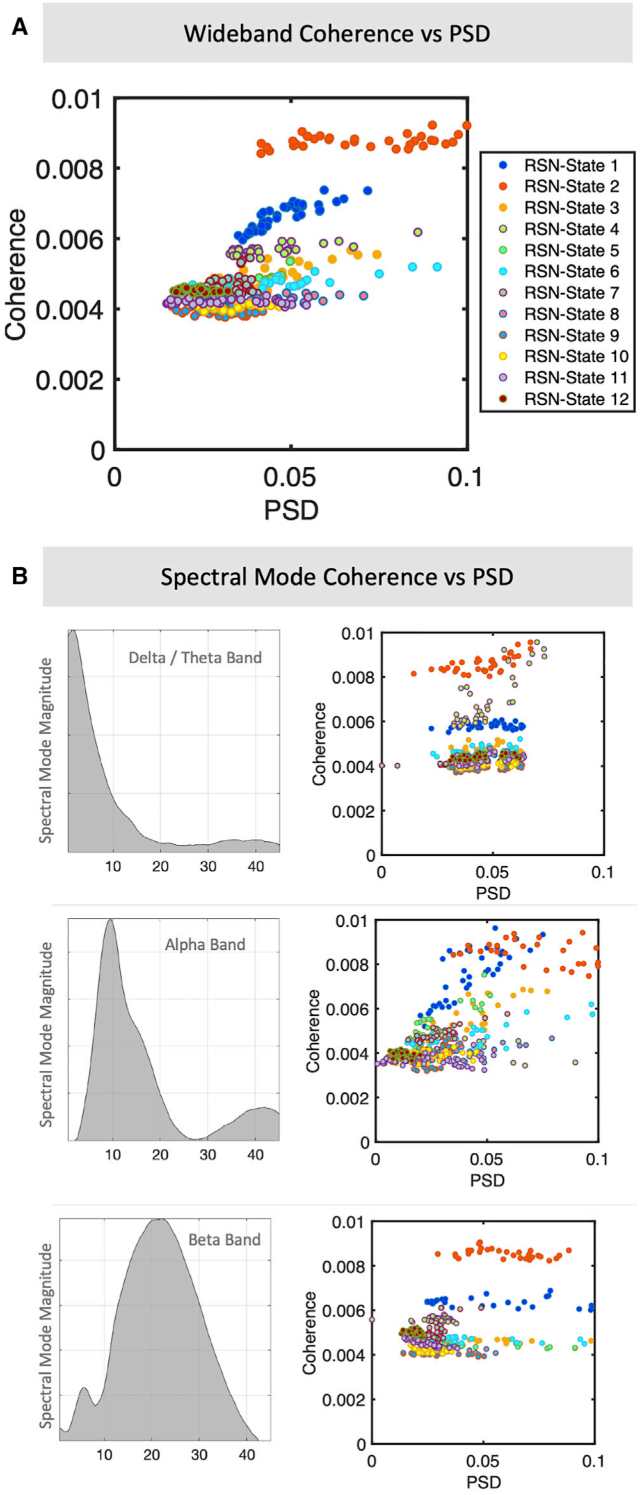

Figure 4. Replay-associated RSN states display specific spectral patterns of power and coherence

(A) Scatterplot of the subject-averaged power spectral density (PSD) and coherence per RSN state, summarizing the overall frequency spectra with a single wide-band average (STAR methods); each point represents one ROI (PSD is computed directly per ROI; coherence is taken as the sum of coherence values between that ROI and all others). This identifies RSN states 1 and 2 by their prominently elevated overall coherence.

(B) The data support a frequency decomposition into three modes that correspond to canonical delta/theta, alpha, and beta bands (STAR methods). Scatterplots of each RSN state's PSD and coherence highlight that RSN state 2 displays higher coherent activity across multiple frequency bands compared with all other RSN states, whereas RSN state 1 is more concentrated in the alpha band.

Figure 6A plots the average high-frequency power spectra over all periods when a given RSN state is active. This demonstrates a significantly elevated power spectral density across all frequencies between 50 and 150 Hz associated exclusively with the DMN RSN state (one-way ANOVA, $p < 2.5e-6$ for each frequency band between 52 and 148 Hz; $p > 0.05$ when ANOVA excludes the DMN). This relationship mimics that observed in the power spectral density averaged over 30-ms windows around replay events (Figure 6B) while accentuating the power increase in much higher frequencies. Plotting the spatial distribution of this high-frequency power in the DMN RSN state (Figure 6C) identifies a concentration of power in temporal regions, areas that encompass the hippocampus and neocortical regions known to couple to ripple activity (Axmacher et al., 2008; Vaz et al., 2019).

Notwithstanding the broadband nature of this signal and the limitations of MEG in imaging deep sources, our results suggest a relationship between RSN state activity and the high-frequency bursts associated with sharp wave ripples, with such bursts occurring exclusively during activation of the DMN RSN state.

Figure S5 demonstrates replication of these results on a second dataset of 22 subjects (Vidaurre et al., 2018; STAR methods) where the DMN was found to be significantly associated with elevated high-frequency power (one-way ANOVA; $p < 2.4e-6$ for all frequencies between 52 Hz and 148 Hz; $p > 0.05$ when the DMN state was omitted), with a spatial distribution of power concentrated over temporal cortices.

Discussion

These results bridge two quite separate fields of enquiry in neuroscience. The study of replay has been predominantly characterized at the level of cellular connections and Local Field Potential (LFP) oscillations in animals, whereas the study of resting brain networks has largely been the preserve of human neuroimaging. Therefore, the link we now establish between these has the potential to extend not only our understanding of replay but also our understanding of the functional role played by human resting brain networks.

The DMN and parietal alpha activity have parallel histories in the scientific literature, initially interpreted as reflecting idling or default patterns of activity and only subsequently understood to have functional roles supporting attention and cognition (Raichle, 2015; Ray and Cole, 1985). The DMN in particular has since been linked to a role broadly defined as internally oriented cognition, encompassing functions such as episodic memory and future-oriented thought (Andrews-Hanna et al., 2010; Raichle, 2015). But in the same way that the brain uses sleep to replay past experience and consolidate memories, our results

CellPress
OPEN ACCESS

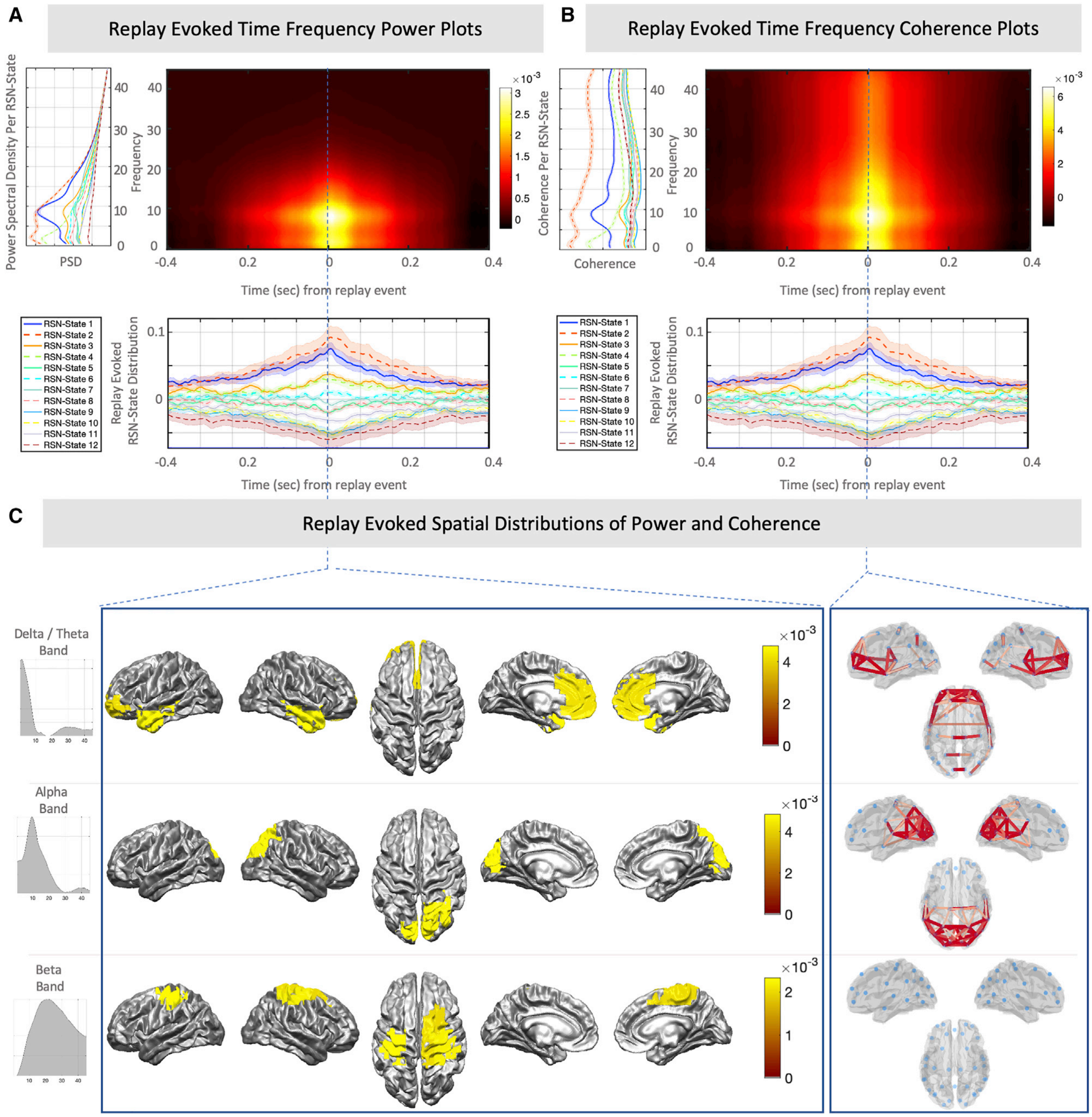

Figure 5. Replay-associated brain activity is characterized by independent spatially and spectrally defined modes

(A and B) Using the replay-evoked RSN state probabilities (bottom panel; mean ± SEM over subjects) as weights for the spectral information unique to each RSN state (left panel; displays average over all ROIs; mean ± SEM over subjects) allows a reconstructed time-frequency estimate of PSD (A) and coherence (B) around replay events, revealing a prominent peak in the alpha and delta/theta bands.

(C) Plotting the spatial distribution of activity in the defined frequency modes at the time of replay identifies independent modes of coherent activity: a low-frequency mode (top panel) comprising the frontal DMN and temporal areas and an alpha frequency mode (center panel) comprising parietal DMN regions and the visual cortex. Additionally, some weaker levels of activity in the beta band are observed over motor areas, but network coherence patterns in this frequency band are not significant (bottom panel).

suggest that healthy waking brain activity may undergo periods of heightened DMN and alpha activity to perform the same function alongside ongoing cognition. Given our more refined mechanistic understanding of the role of replay, our new findings could extend our interpretation of the functional relevance of the DMN. Replay itself is fundamentally understood as a mechanism for

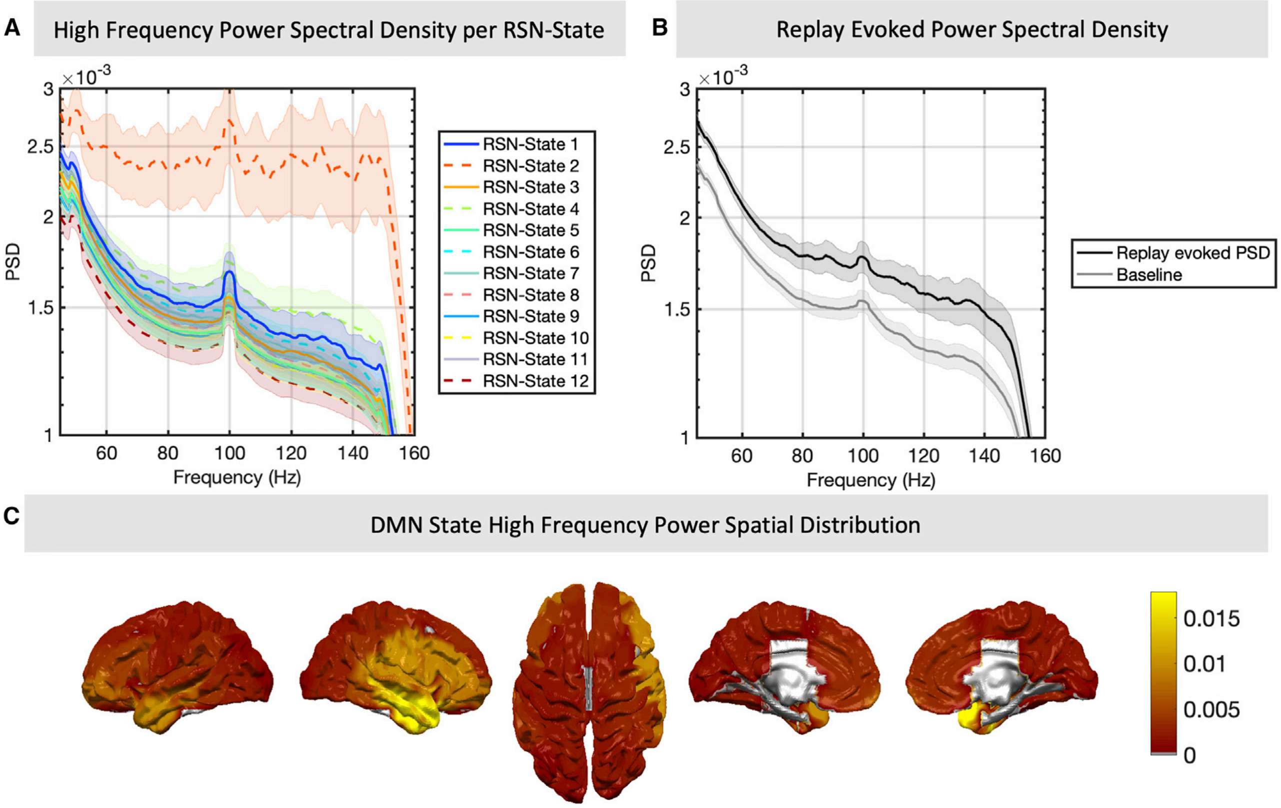

Figure 6. Replay and the DMN coincide with high-frequency power increases in temporal areas

(A) Although the RSN state model was originally fit to data filtered at 1–45 Hz, we can still analyze whether the state timings correlate with specific patterns in frequency bands outside of this range in the original data. This reveals a very strong association between RSN state 2 and power in high frequencies (mean ± SEM over subjects) despite these high frequencies not having been included originally in the model.

(B) Similarly, the onset of replay is associated with an increase in high-frequency power relative to the global average (mean ± SEM over subjects). Replay-evoked power was computed by taking 100-ms windows of data around identified replay events; for baseline, see STAR methods.

(C) The spatial distribution of high-frequency (102–148 Hz) power in the DMN state; activity in this RSN state and in this frequency band source localizes to the temporal cortex.

memory consolidation but has also been proposed to have more expansive roles in building cognitive maps, preparing neural structures for learning (preplay), and transferring knowledge from the hippocampus to the cortex (Dragoi and Tonegawa, 2011; Foster, 2017). This suggests a more expansive role of the DMN in executive control, with the regular transient activations of the DMN associated with building and maintaining stable representations of recently acquired information.

Replay occurs at highest intensity during slow-wave sleep, when large-scale synchronized oscillations provide an environment conducive to large-scale propagation of sharp wave ripples (Sirota et al., 2003). Notably, the RSN states that correlate with replay in our study are characterized by large increases in oscillatory coherence, itself hypothesized to support integration of signals from disparate regions of the cortex. The DMN itself appears to be preserved at least into light sleep stages (Greicius et al., 2008; Larson-Prior et al., 2009; Sämann et al., 2011), with further evidence showing that the DMN correlates directly with sharp wave ripples under light anesthesia (Kaplan et al., 2016). Our findings appear to reflect both of these phenomena, low-frequency coherent oscillations coupled to high-frequency power bursts, coinciding directly with estimated replay events during an awake state. Such high-frequency power bursts are taken to reflect increased aggregate rates of action potential discharge (Miller et al., 2009), which may or may not coincide with sharp wave ripples. Importantly, the high-frequency band power increases we see alongside DMN activation appear to be much more broadband than what has been established by studies of human sharp wave ripple activity using invasive methods. We do not have a good explanation for this at present, but it is notable that comparable broadband spectra have been detected by studies analyzing ripple band activity in neocortical areas locked to hippocampal ripple events (Axmacher et al., 2008; Helfrich et al., 2019; Jiang et al., 2017; Norman et al., 2019; Vaz et al., 2019). Furthermore, the replay-evoked spectrum in higher frequencies appears to be more narrowband when baseline correction is applied, indicating that this narrowband/broadband distinction may be sensitive to such methodological choices (Figure S5).

The role of the parietal alpha network is perhaps more readily understood through the unique requirements of awake-state replay. Our results are based on spontaneous

CellPress
OPEN ACCESS

replay of visual stimulus representations, raising the problem of how the brain could reinstate these items without interference from ongoing perception. Alpha oscillations are widely interpreted as an inhibitory signal that acts to gate irrelevant stimuli from active processing (Foxe and Snyder, 2011; Jensen and Mazaheri, 2010; Rihs et al., 2007). One possibility is that strong alpha activity may combine with DMN activation to inhibit bottom-up sensory perception during inward-oriented attention, supporting replay of items from memory. This could provide a crucial mechanism for how replay plays out without interference from competing sensory inputs during the awake state.

We have focused on sequential reactivations consistent with task sequence, but similar analyses work when we simply consider all reactivations (Figure S6). It has been established in these datasets that reactivations are more likely to occur in relevant sequences than in control sequences (Liu et al., 2019). However, because reactivations are bursty, we cannot make selective claims about sequential reactivations here. Increases in the density of sequential reactivations coincide with increases in the density of all reactivations, and these times align with periods of DMN and parietal alpha activity.

In our work, we have drawn a clear distinction between spontaneous replay and cued reactivations but acknowledge that it is not clear how important this distinction might be in general. Notably, a number of MEG and EEG studies that have defined replay differently in the context of cued memory recall paradigms (Jafarpour et al., 2013; Kerrén et al., 2018; Michelmann et al., 2016, 2019) or event boundary analyses (Sols et al., 2017) have reported findings that appear to overlap with our own; in particular, finding strong dependencies on neural dynamics in the theta/alpha frequency range (Kerrén et al., 2018; Michelmann et al., 2016, 2019). In the broader context of our findings, this might suggest common neural mechanisms for cued memory recall and spontaneous replay, mechanisms that may be a core function of the DMN.

The temporal profile of replay activation that we have characterized may also explain replay-related signals across different recording modalities. In particular, fMRI studies have shown reliable behavioral correlations between the reinstatement of blood-oxygen-level-dependent (BOLD) traces associated with experimental stimuli and subsequent task performance (Deuker et al., 2013; Momennejad et al., 2018; Tambini and Davachi, 2019). This has supported an interpretation of the BOLD signal as a reflection of cellular replay despite the disparity of timescales between cellular replay events (known to be temporally compressed on the order of milliseconds) and the hemodynamic response (assumed to reflect sustained activations on the order of seconds). However, our results characterize replay as occurring in transient bursts of high intensity interspersed with long periods of quiescence. Such a temporal profile may bridge this disparity of timescales and explain how a hemodynamic signal could arise from clusters of replay bursts in quick succession.

Furthermore, our results can help to bridge the understanding of RSNs studied in electrophysiology and fMRI. A long-standing challenge in unifying findings across modalities has been to understand how the BOLD response relates to activity in canonical frequency bands. Activity in the gamma band (>30 Hz) has consistently shown a strong correlation with a subsequent BOLD signal (Niessing et al., 2005; Nir et al., 2007); however, a more complex relationship emerges between the BOLD signal and activity in lower-frequency bands, in which electrophysiological RSNs are largely defined (Brookes et al., 2011; Mantini et al., 2007). It has now been shown that different RSN states show markedly different hemodynamic profiles; in particular, the DMN and DAN evoke BOLD signals that are opposed in polarity and distinct in their temporal decay profile (Sitnikova et al., 2020). If the DMN state visits cluster together in time while being linked to high-frequency power increases, as our results indicate, then this may explain these distinct profiles and provide a key bridge between the understanding of RSNs recorded across distinct modalities.

Finally, our results suggest measures that could potentially serve as non-invasive proxy measures of replay, potentially opening the door to a broader set of replay experimental paradigms. Until very recently, replay has been predominantly studied in animal models using spatial navigation paradigms because of the necessity of highly invasive electrophysiology to detect replay and the sophisticated understanding of the entorhinal-hippocampal spatial navigation systems. The development of methods to detect spontaneous reactivation (Tambini and Davachi, 2019) and replay (Kurth-Nelson et al., 2016; Liu et al., 2019; Schuck and Niv, 2019) in humans noninvasively has enabled experiments that test how these theories generalize to non-spatial domains and other abstract cognitive tasks that are unique to human neuroscience. However, these methods require demanding experimental designs and cognitive paradigms. Our results further broaden the range of tools, providing additional non-invasive measures that could provide an estimate of aggregate replay activity under simple experimental conditions (rest) that can be potentially be studied in large populations or patient groups.

Overall, our results highlight an important link between two influential domains of research in modern neuroscience, the study of replay and the study of RSNs, and provide a potential connection between noninvasive human imaging studies and invasive cellular physiology.

STAR★METHODS

Detailed methods are provided in the online version of this paper and include the following:

- KEY RESOURCES TABLE
- RESOURCE AVAILABILITY
  - Lead contact
  - Materials availability
  - Data and code availability
- EXPERIMENTAL MODEL AND SUBJECT DETAILS
- METHOD DETAILS
  - Replay Experimental protocol
  - Replay Detection
  - Resting State Network Modeling
- QUANTIFICATION AND STATISTICAL ANALYSIS
  - MEG Data Acquisition
  - Data Preprocessing
  - Source Space Reconstruction

- ○ Model inference
- ○ Model Parameter Selection
- ○ RSN-state labeling
- ○ RSN Spectral Information
- ○ Replay Evoked RSN Analysis
- ○ Controlling for Replay Classifier Variance During DMN Activation
- ○ Comparing Reactivation Evoked and Replay Evoked Distributions
- ○ Spectral Characterizations of Replay and Classifier Outputs
- ○ Transient Burst Activity Analysis
- ○ RSN-state Power and Coherence Analysis
- ○ Replay Evoked Spatial Maps
- ○ High Frequency Oscillations
- ○ Baseline Correction for High Frequency Oscillations

Supplemental Information

Supplemental Information can be found online at https://doi.org/10.1016/j.neuron.2020.12.007.

Acknowledgments

We are grateful to Andrew Quinn for guidance regarding data processing, beamforming, and visualization code and Jiewon Kang for preliminary investigations of high-frequency oscillations. Both Wellcome Centres are supported by core funding from the Wellcome Trust: the Wellcome Centre for Integrative Neuroimaging (203139/Z/16/Z) and the Wellcome Trust Centre for Human Neuroimaging (091593/Z/10/Z). M.W.'s research is supported by the NIHR Oxford Health Biomedical Research Centre, the Wellcome Trust (106183/Z/14/Z and 215573/Z/19/Z), and the New Therapeutics in Alzheimer's Diseases (NTAD) study supported by UK MRC and the Dementia Platform UK. T.B. was supported by Wellcome Trust senior (104765/Z/14/Z) and principal (219525/Z/19/Z) research fellowships and a Wellcome collaborator award (214314/Z/18/Z) during the course of the research. R.D. is supported by a Wellcome Trust investigator award (098362/Z/12/Z). This work was carried out whilst R.D. was in receipt of a Lundbeck visiting professorship (R290-2018-2804) to the Danish Research Centre for Magnetic Resonance. Y.L. is supported by a UCL graduate research scholarship and a UCL overseas research scholarship. Y.L. is a pre-doctoral fellow of the International Max Planck Research School on Computational Methods in Psychiatry and Ageing Research (IMPRS COMP2PSYCH). The UCL-Max Planck Centre for Computational Psychiatry and Ageing is funded by a joint initiative between UCL and the Max Planck Society. D.V. is supported by a Novo Nordisk Foundation grant (NNF19OC-005489).

Author contributions

Y.L., C.H., R.D., Z.K.-N., M.W., and T.B. conceptualized the research goals. M.W. and T.B. supervised the investigation. D.V., M.W., and C.H. developed the methodology and software implementation. Y.L. collected and curated the data and assisted with formal data analysis. C.H. formally analyzed the data and wrote the initial draft. All authors reviewed and edited the manuscript.

Declaration of interests

Z.K.-N. is employed by DeepMind Technologies Limited.

Received: July 3, 2020
Revised: October 15, 2020
Accepted: December 4, 2020
Published: December 23, 2020

References

Andrews-Hanna, J.R., Reidler, J.S., Huang, C., and Buckner, R.L. (2010). Evidence for the default network's role in spontaneous cognition. J. Neurophysiol. *104*, 322–335.

Axmacher, N., Elger, C.E., and Fell, J. (2008). Ripples in the medial temporal lobe are relevant for human memory consolidation. Brain *131*, 1806–1817.

Baker, A.P., Brookes, M.J., Rezek, I.A., Smith, S.M., Behrens, T., Probert Smith, P.J., and Woolrich, M. (2014). Fast transient networks in spontaneous human brain activity. eLife *3*, e01867.

Biswal, B., Yetkin, F.Z., Haughton, V.M., and Hyde, J.S. (1995). Functional connectivity in the motor cortex of resting human brain using echo-planar MRI. Magn. Reson. Med. *34*, 537–541.

Brookes, M.J., Woolrich, M., Luckhoo, H., Price, D., Hale, J.R., Stephenson, M.C., Barnes, G.R., Smith, S.M., and Morris, P.G. (2011). Investigating the electrophysiological basis of resting state networks using magnetoencephalography. Proc. Natl. Acad. Sci. USA *108*, 16783–16788.

Buzsáki, G. (1998). Memory consolidation during sleep: a neurophysiological perspective. J. Sleep Res. *7* (*Suppl 1*), 17–23.

Buzsáki, G. (2015). Hippocampal sharp wave-ripple: A cognitive biomarker for episodic memory and planning. Hippocampus *25*, 1073–1188.

Carr, M.F., Jadhav, S.P., and Frank, L.M. (2011). Hippocampal replay in the awake state: a potential substrate for memory consolidation and retrieval. Nat. Neurosci. *14*, 147–153.

Chrobak, J.J., and Buzsáki, G. (1996). High-frequency oscillations in the output networks of the hippocampal-entorhinal axis of the freely behaving rat. J. Neurosci. *16*, 3056–3066.

Colclough, G.L., Brookes, M.J., Smith, S.M., and Woolrich, M.W. (2015). A symmetric multivariate leakage correction for MEG connectomes. Neuroimage *117*, 439–448.

Colclough, G.L., Woolrich, M.W., Tewarie, P.K., Brookes, M.J., Quinn, A.J., and Smith, S.M. (2016). How reliable are MEG resting-state connectivity metrics? Neuroimage *138*, 284–293.

de Pasquale, F., Della Penna, S., Snyder, A.Z., Lewis, C., Mantini, D., Marzetti, L., Belardinelli, P., Ciancetta, L., Pizzella, V., Romani, G.L., and Corbetta, M. (2010). Temporal dynamics of spontaneous MEG activity in brain networks. Proc. Natl. Acad. Sci. USA *107*, 6040–6045.

Deuker, L., Olligs, J., Fell, J., Kranz, T.A., Mormann, F., Montag, C., Reuter, M., Elger, C.E., and Axmacher, N. (2013). Memory consolidation by replay of stimulus-specific neural activity. J. Neurosci. *33*, 19373–19383.

Dragoi, G., and Tonegawa, S. (2011). Preplay of future place cell sequences by hippocampal cellular assemblies. Nature *469*, 397–401.

Foster, D.J. (2017). Replay comes of age. Annu. Rev. Neurosci. *40*, 581–602.

Fox, M.D., and Raichle, M.E. (2007). Spontaneous fluctuations in brain activity observed with functional magnetic resonance imaging. Nat. Rev. Neurosci. *8*, 700–711.

Foxe, J.J., and Snyder, A.C. (2011). The role of alpha-band brain oscillations as a sensory suppression mechanism during selective attention. Front. Psychol. *2*, 154.

Fukunaga, M., Horovitz, S.G., van Gelderen, P., de Zwart, J.A., Jansma, J.M., Ikonomidou, V.N., Chu, R., Deckers, R.H.R., Leopold, D.A., and Duyn, J.H. (2006). Large-amplitude, spatially correlated fluctuations in BOLD fMRI signals during extended rest and early sleep stages. Magn. Reson. Imaging *24*, 979–992.

Greicius, M.D., Kiviniemi, V., Tervonen, O., Vainionpää, V., Alahuhta, S., Reiss, A.L., and Menon, V. (2008). Persistent default-mode network connectivity during light sedation. Hum. Brain Mapp. *29*, 839–847.

Helfrich, R.F., Lendner, J.D., Mander, B.A., Guillen, H., Paff, M., Mnatsakanyan, L., Vadera, S., Walker, M.P., Lin, J.J., and Knight, R.T. (2019). Bidirectional prefrontal-hippocampal dynamics organize information transfer during sleep in humans. Nat. Commun. *10*, 3572.

CellPress
OPEN ACCESS

Huang, M.X., Mosher, J.C., and Leahy, R.M. (1999). A sensor-weighted overlapping-sphere head model and exhaustive head model comparison for MEG. Phys. Med. Biol. *44*, 423–440.

Hunt, B.A.E., Tewarie, P.K., Mougin, O.E., Geades, N., Jones, D.K., Singh, K.D., Morris, P.G., Gowland, P.A., and Brookes, M.J. (2016). Relationships between cortical myeloarchitecture and electrophysiological networks. Proc. Natl. Acad. Sci. USA *113*, 13510–13515.

Jafarpour, A., Horner, A.J., Fuentemilla, L., Penny, W.D., and Duzel, E. (2013). Decoding oscillatory representations and mechanisms in memory. Neuropsychologia *51*, 772–780.

Jensen, O., and Mazaheri, A. (2010). Shaping functional architecture by oscillatory alpha activity: gating by inhibition. Front. Hum. Neurosci. *4*, 186.

Ji, D., and Wilson, M.A. (2007). Coordinated memory replay in the visual cortex and hippocampus during sleep. Nat. Neurosci. *10*, 100–107.

Jiang, X., Shamie, I., K Doyle, W., Friedman, D., Dugan, P., Devinsky, O., Eskandar, E., Cash, S.S., Thesen, T., and Halgren, E. (2017). Replay of large-scale spatio-temporal patterns from waking during subsequent NREM sleep in human cortex. Sci. Rep. *7*, 17380.

Kaplan, R., Adhikari, M.H., Hindriks, R., Mantini, D., Murayama, Y., Logothetis, N.K., and Deco, G. (2016). Hippocampal sharp-wave ripples influence selective activation of the default mode network. Curr. Biol. *26*, 686–691.

Kerrén, C., Linde-Domingo, J., Hanslmayr, S., and Wimber, M. (2018). An Optimal Oscillatory Phase for Pattern Reactivation during Memory Retrieval. Curr. Biol. *28*, 3383–3392.e6.

Kudrimoti, H.S., Barnes, C.A., and McNaughton, B.L. (1999). Reactivation of hippocampal cell assemblies: effects of behavioral state, experience, and EEG dynamics. J. Neurosci. *19*, 4090–4101.

Kurth-Nelson, Z., Economides, M., Dolan, R.J., and Dayan, P. (2016). Fast sequences of non-spatial state representations in humans. Neuron *91*, 194–204.

Larson-Prior, L.J., Zempel, J.M., Nolan, T.S., Prior, F.W., Snyder, A.Z., and Raichle, M.E. (2009). Cortical network functional connectivity in the descent to sleep. Proc. Natl. Acad. Sci. USA *106*, 4489–4494.

Liu, Y., Dolan, R.J., Kurth-Nelson, Z., and Behrens, T.E.J. (2019). Human replay spontaneously reorganizes experience. Cell *178*, 640–652.e14.

Liu, Y., Dolan, R.J., Penagos-Vargas, H.L., Kurth-Nelson, Z., and Behrens, T. (2020). Measuring Sequences of Representations with Temporally Delayed Linear Modelling. bioRxiv. https://doi.org/10.1101/2020.04.30.066407.

Mantini, D., Perrucci, M.G., Del Gratta, C., Romani, G.L., and Corbetta, M. (2007). Electrophysiological signatures of resting state networks in the human brain. Proc. Natl. Acad. Sci. USA *104*, 13170–13175.

Michelmann, S., Bowman, H., and Hanslmayr, S. (2016). The Temporal Signature of Memories: Identification of a General Mechanism for Dynamic Memory Replay in Humans. PLoS Biol. *14*, e1002528.

Michelmann, S., Staresina, B.P., Bowman, H., and Hanslmayr, S. (2019). Speed of time-compressed forward replay flexibly changes in human episodic memory. Nat. Hum. Behav. *3*, 143–154.

Miller, K.J., Sorensen, L.B., Ojemann, J.G., and den Nijs, M. (2009). Power-law scaling in the brain surface electric potential. PLoS Comput. Biol. *5*, e1000609.

Momennejad, I., Otto, A.R., Daw, N.D., and Norman, K.A. (2018). Offline replay supports planning in human reinforcement learning. eLife *7*, e32548.

Niessing, J., Ebisch, B., Schmidt, K.E., Niessing, M., Singer, W., and Galuske, R.A.W. (2005). Hemodynamic signals correlate tightly with synchronized gamma oscillations. Science *309*, 948–951.

Nir, Y., Fisch, L., Mukamel, R., Gelbard-Sagiv, H., Arieli, A., Fried, I., and Malach, R. (2007). Coupling between neuronal firing rate, gamma LFP, and BOLD fMRI is related to interneuronal correlations. Curr. Biol. *17*, 1275–1285.

Norman, Y., Yeagle, E.M., Khuvis, S., Harel, M., Mehta, A.D., and Malach, R. (2019). Hippocampal sharp-wave ripples linked to visual episodic recollection in humans. Science *365*, 1–14.

Qin, Y.L., McNaughton, B.L., Skaggs, W.E., and Barnes, C.A. (1997). Memory reprocessing in corticocortical and hippocampocortical neuronal ensembles. Philos. Trans. R. Soc. Lond. B Biol. Sci. *352*, 1525–1533.

Quinn, A.J., Vidaurre, D., Abeysuriya, R., Becker, R., Nobre, A.C., and Woolrich, M.W. (2018). Task-Evoked Dynamic Network Analysis Through Hidden Markov Modeling. Front. Neurosci. *12*, 603.

Raichle, M.E. (2015). The brain's default mode network. Annu. Rev. Neurosci. *38*, 433–447.

Raichle, M.E., MacLeod, A.M., Snyder, A.Z., Powers, W.J., Gusnard, D.A., and Shulman, G.L. (2001). A default mode of brain function. Proc. Natl. Acad. Sci. USA *98*, 676–682.

Ray, W.J., and Cole, H.W. (1985). EEG alpha activity reflects attentional demands, and beta activity reflects emotional and cognitive processes. Science *228*, 750–752.

Rihs, T.A., Michel, C.M., and Thut, G. (2007). Mechanisms of selective inhibition in visual spatial attention are indexed by α-band EEG synchronization. Eur. J. Neurosci. *25*, 603–610.

Sämann, P.G., Wehrle, R., Hoehn, D., Spoormaker, V.I., Peters, H., Tully, C., Holsboer, F., and Czisch, M. (2011). Development of the brain's default mode network from wakefulness to slow wave sleep. Cereb. Cortex *21*, 2082–2093.

Schuck, N.W., and Niv, Y. (2019). Sequential replay of nonspatial task states in the human hippocampus. Science *364*, 1–9.

Sirota, A., Csicsvari, J., Buhl, D., and Buzsáki, G. (2003). Communication between neocortex and hippocampus during sleep in rodents. Proc. Natl. Acad. Sci. USA *100*, 2065–2069.

Sitnikova, T., Hughes, J.W., Howard, C.M., Stephens, K.A., Woolrich, M., and Salat, D.H. (2020). Spontaneous activity changes in large-scale cortical networks in older adults couple to distinct hemodynamic morphology. bioRxiv. https://doi.org/10.1101/2020.05.05.079749.

Sols, I., DuBrow, S., Davachi, L., and Fuentemilla, L. (2017). Event Boundaries Trigger Rapid Memory Reinstatement of the Prior Events to Promote Their Representation in Long-Term Memory. Curr. Biol. *27*, 3499–3504.e4.

Tambini, A., and Davachi, L. (2019). Awake reactivation of prior experiences consolidates memories and biases cognition. Trends Cogn. Sci. *23*, 876–890.

Teich, M.C., Turcott, R.G., and Siegel, R.M. (1996). Temporal correlation in cat striate-cortex neural spike trains. IEEE Eng. Med. Biol. Mag. *15*, 79–87.

Vaz, A.P., Inati, S.K., Brunel, N., and Zaghloul, K.A. (2019). Coupled ripple oscillations between the medial temporal lobe and neocortex retrieve human memory. Science *363*, 975–978.

Vidaurre, D., Quinn, A.J., Baker, A.P., Dupret, D., Tejero-Cantero, A., and Woolrich, M.W. (2016). Spectrally resolved fast transient brain states in electrophysiological data. Neuroimage *126*, 81–95.

Vidaurre, D., Hunt, L.T., Quinn, A.J., Hunt, B.A.E., Brookes, M.J., Nobre, A.C., and Woolrich, M.W. (2018). Spontaneous cortical activity transiently organises into frequency specific phase-coupling networks. Nat. Commun. *9*, 2987.

Wilson, M.A., and McNaughton, B.L. (1994). Reactivation of hippocampal ensemble memories during sleep. Science *265*, 676–679.

Woolrich, M., Hunt, L., Groves, A., and Barnes, G. (2011). MEG beamforming using Bayesian PCA for adaptive data covariance matrix regularization. Neuroimage *57*, 1466–1479.

STAR★METHODS

KEY RESOURCES TABLE

REAGENT or RESOURCE	SOURCE	IDENTIFIER
Software and algorithms		
HMM-MAR toolbox	Vidaurre et. al., 2018	https://github.com/OHBA-analysis/HMM-MAR
OHBA Software Library	Woolrich et. al., 2011	https://ohba-analysis.github.io/osl-docs/pages/overview/download.html
Custom code for this paper	N/A	https://github.com/OHBA-analysis/Higgins2020_Neuron

Resource availability

Lead contact

Further information and requests for resources should be directed to Cameron.Higgins@ohba.ox.ac.uk.

Materials availability

This study did not generate any new reagents.

Data and code availability

The code used to run the analyses in this paper is publicly available at the following GitHub repository: https://github.com/OHBA-analysis/Higgins2020_Neuron

This paper analyses data from three separate datasets, referred to as Dataset A, B and C (see STAR methods). Dataset A and B will be freely available upon request (subject to participant consent) to yunzhe.liu.16@ucl.ac.uk. Dataset C was collected and held by the MEG UK Partnership, and access to this data can be requested at https://meguk.ac.uk/contact

Experimental model and subject details

We make reference throughout this publication to three separate datasets; we will refer to these as Dataset A, the replay data collected by Liu et al. (2019) that forms the main focus of analysis in this paper; Dataset B, a secondary replay dataset that was used to prove replication of the analysis conducted on dataset A, which is referred to throughout the paper and is the focus of Figures S2–S5; and Dataset C, an established, larger dataset comprising resting state MEG data that we used to train the canonical resting state networks.

Dataset A, the primary replay dataset, was acquired from 25 participants (aged 19-34, mean age 24.89; eleven male, two left-handed); four subjects were then excluded due to large motion artifacts or missing trigger information, leaving 21 subjects for the analysis conducted. All participants signed written consent in advance; ethical approval for the experiment was obtained from the Research Ethics Committee at University College London under ethics number 9929/002.

Dataset B, the replication dataset, was acquired from 26 participants (aged 19-34, mean age 25.48; ten male, two left-handed); four participants were later excluded due to motion artifacts or failure to complete the task, leaving 22 subjects for the analysis conducted. All participants signed written consent in advance; ethical approval for the experiment was obtained from the Research Ethics Committee at University College London under ethics number 9929/002.

Dataset C, the RSN-state training dataset, comprised resting state MEG data from a larger group of subjects that has previously been used to characterize MEG resting state network dynamics (Hunt et al., 2016; Vidaurre et al., 2018). This study acquired resting state MEG scans and structural MRI scans for 55 participants (mean age 26.5 years, maximum age 48 years, minimum age 18 years; 35 males). All participants gave written informed consent and ethical approval was granted by the University of Nottingham Medical School Research Ethics Committee.

METHOD DETAILS

Replay Experimental protocol

For full details of the experimental protocol, readers are directed to Liu et. al. 2019; key details only are summarized here. In Dataset A, each participant attended two days, the first for learning the structure of the task and the second for completing the task while undergoing MEG scanning. The task was based around 8 visual stimuli, and participant's objective was to correctly unshuffle these

CellPress
OPEN ACCESS

into the two correct four item-long sequences (eg, A- > B- > C- > D and A' - > B' - > C' - > D'), using a set of unshuffling rules learned on the first day of the experiment. Once in the scanner, participants observed multiple presentations of the novel visual stimuli in a randomized order to act as training data for the multivariate classifiers (the Functional Localizer data. They were then presented with the visual stimuli in the shuffled order from which they could infer the correct sequence. Participants then underwent the first 5 minute resting state scan; one of the two four-item sequences was paired with a reward; participants underwent a second resting state scan, and were tested on their correct recall of the item sequences. All analysis in this paper focuses only on the data from the two resting state scans within this overall experiment. Dataset B used a very similar but slightly amended paradigm; the shuffling rule that participants learned was different, as was the ordering of individual blocks (see Liu et. al. 2019).

Replay Detection

This paper builds on the results of Liu et al. (2019), using their computed replay onset timings. For clarity we outline their methods to detect replay here, but direct readers to the original publication for further details. For each of the eight visual stimuli, sparse logistic regression classifiers were trained using L1 regularization on the functional localizer data to identify the neural patterns associated with each visual stimulus. To select an appropriate time point on which to train the classifiers, the authors used cross validation over the functional localizer data to plot the overall classification accuracy; this identified the time point 200msec following stimulus presentation to be the time point corresponding to the highest classification accuracy averaged over all stimuli, trials and subjects. The trained classifiers associated with this time point were then fit to the resting state data, producing eight time series that represented the probability of reinstatement of the activity patterns associated with each visual stimulus:

$$A_t = \sigma(X_t\beta_A)$$

$$B_t = \sigma(X_t\beta_B)$$

Where X_t is the recorded resting state data at time t; β_i is the sparse classifier coefficients associated with the ith visual stimulus; A_t is the probability of reinstatement of the patterns associated with stimulus A at time t in the resting state scan; and $\sigma(x)$ denotes the logistic sigmoid transform which maps from the real number plane to a probability on the interval [0,1].

The authors analyzed the temporal cross-correlation of these scores and found significant evidence for the reinstatement of stimulus patterns in specific sequence ordering that matched the task structure; that is, scores followed in the patterns A- > B- > C- > D and A'- > B'- > C'- > D' (see Figure 1B). This patterning was strongest for a time lag of $\tau = 40$msec, indicating very rapid serial reinstatement of visual stimulus representations. Finally, the authors estimated a single overall time course of replay R_t using the following logical operation:

$$R_t = P\begin{pmatrix} (A_t \cap B_{t+\tau}) \cup (B_t \cap C_{t+\tau}) \cup (C_t \cap D_{t+\tau}) \\ \cup (A_t{}' \cap B_{t+\tau}{}') \cup (B_t{}' \cap C_{t+\tau}{}') \cup (C_t{}' \cap D_{t+\tau}{}') \end{pmatrix}$$

Where $\cap$ denotes the logical AND operation, and $\cup$ denotes the logical OR operation. Consequently, the replay time course, R_t, represents the probability of any task-relevant two item sequence occurring with the specified time lag $\tau = 40$msec. This probabilistic output was thresholded at the 99th percentile to provide the estimated replay event times used throughout this paper.

Resting State Network Modeling

The RSN-states referred to in Figure 1 and throughout this paper use an established Hidden Markov Model with Time Delay Embedding (HMM-TDE) approach outlined in detail in Vidaurre et al. (2018). We outline the approach here and direct readers to the original publication for further details.

This model makes use of the Hidden Markov Modeling framework, a generative modeling approach that describes the data X_t at each time point t as being generated from a latent state variable $Z_t \in [1, 2, \ldots K]$. That is, the latent state at any point in time is an integer between 1 and K, where K is a parameter controlling the total number of states. Therefore, the DMN state being active at time t would be denoted by $Z_t = 2$, with the DMN corresponding to RSN-state 2 in this case (Figure 2B).

To complete the model specification, we must define the observation model – that is, the probabilistic model relating the activation of a particular latent state to the underlying data. As in Vidaurre et al. (2018), we make use of a temporal embedding with a Gaussian observation model:

$$P(vec(X_{t-l:t+l}) \mid Z_t = k) \sim N(0, \Sigma_k)$$

In this notation, the vec operator performs the temporal embedding; that is, we take a $[W \times P]$ matrix of points $X_{t-l:t+l}$ centered on the time point t, where P is the number of data dimensions and $W = 2l + 1$ is the length of the temporal embedding - and stack it into a $[WP \times 1]$ vector with the vec operator. We then model this vector of datapoints with a Gaussian distribution, with zero mean and a $[WP \times WP]$ covariance matrix determined by the active state. Crucially, each RSN-state is now parameterized by a unique covariance matrix which reflects the autocovariance on each channel, as well as the cross covariance across channels. As outlined in Vidaurre et al. (2018), this is an efficient parameterization of the power spectrum and the cross power spectrum respectively. Consequently, each distinct RSN-state corresponds to a distinct distribution of power on each channel and coherence between channels.

Quantification and statistical analysis

MEG Data Acquisition

In both Dataset A and B, MEG scans were acquired at 600 samples/second on a 275 channel CTF MEG system. For Dataset C, MEG data was acquired at 1200Hz using a 275 channel CTF MEG system operating at third order synthetic gradiometry configuration; MRI data were acquired using a Phillips Achieva 7T system. MRI data, used only for the purpose of MEG coregistration, were acquired using a Philips Achieva 7T scanner.

Data Preprocessing

Across the three datasets and the two analysis pipelines of Figure 1B we sought to minimize differences in preprocessing however minor deviations were necessary. The replay identification conducted by Liu et al. (2019) (Figure 1) filtered sensor space MEG data to a pass band of 0.5 to 50 Hz; data were downsampled to 100 samples / second. All analysis reported by Liu et al. (2019) in this paper was conducted in sensor space; for further details see Liu et al. (2019). The Resting State Network analysis pipeline filtered sensor space data to a pass band of 1 to 45 Hz and downsampled data to 250 samples / second. This slightly amended filter passband was to ensure RSN-state dynamics were not driven by low frequency sensor drift effects or mainline power noise effects, which the HMM modeling approach is more sensitive to compared to the replay identification methods introduced by Liu et al. (2019). Similarly, the higher sampling rate was to ensure sufficient resolution of the time embedding to ensure good estimation of spectral content for each RSN-state definition. The only exception to this was for the final analysis of spectral content in higher frequencies (Figure 6), for which we returned to the raw data to utilize the highest frequency information available; this data had been acquired at 600 samples / second and low pass filtered with cutoff frequency 160Hz.

Source Space Reconstruction

One of the motivations for using Dataset C to train the resting state networks was to ensure the highest possible confidence and replicability of the anatomical distributions of activity unique to each RSN-state. Given the limited spatial resolution of MEG, confidence can be increased through the use of larger datasets which have been coregistered using MRI acquired structural scans. MRI scans were not acquired for either dataset A or dataset B, motivating our focus on dataset C to determine high confidence spatial topographies of each RSN-state.

Dataset C was coregistered to MRI structural information using a multiple local sphere forward model (Huang et al., 1999). Datasets A and B, which did not have associated MRI structural information, were coregistered using fiducial markers. All data then followed a common pipeline of analysis thereafter, with source space reconstruction performed using an LCM-V beamformer projecting to an 8mm MNI grid. This grid was then parcellated into 38 anatomically defined Regions of Interest (ROIs) derived from an independent component analysis of fMRI resting state data from the Human Connectome Project (Colclough et al., 2016). Source leakage was then corrected for by orthogonalization as outlined in (Colclough et al., 2015).

Model inference

The full model outlined above is amenable to variational Bayesian methods, which support fast scalable inference for large datasets. The datasets we have used are moderately large – the full model was trained on dataset C, comprising 5 minutes of resting state scans from 55 subjects at 250samples/second, totaling 3.9 million unique data samples. This defined our set of canonical RSN-states, which we then fixed and subsequently fit to datasets A and B, each comprising a total of 10 minutes of resting state scans from 21 and 22 subjects respectively, totaling 3.2 million unique samples and 3.7 million unique samples, respectively. To enable scaleable inference over such datasets, we utilized stochastic gradient variational Bayes methods that iteratively learn a full model through batch training (Vidaurre et al., 2018). Furthermore, we use PCA dimensionality reduction steps as outlined in Vidaurre et al. (2018) when training the model; to avoid potential misrepresentation of the data, the dimensionality reduced data is only used for model training, all spectral features plotted in figures are based on a multitaper fit back to the original, full dimensional data. Finally, hierarchical models fit with variational methods can be sensitive to local minima. To ensure good convergence, the full model inference was run five times; only the model with the lowest final free energy was kept, the others were discarded.

Model Parameter Selection

As a guiding principle we have maintained the same parameter choice as that previously used in (Vidaurre et al., 2018). The parameter K controls the total number of RSN-states and therefore the granularity of the solution; we set this to $K = 12$, consistent with previous publications; the same results can be broadly reproduced with other choices of K.

RSN-state labeling

The HMM-TDE model infers a set of mutually exclusive latent states, which we number from 1 to 12 for ease of reference throughout. The RSN-state numbers themselves are arbitrary, however for ease of interpretation we assign labels using a data-driven approach that minimizes a distance metric between ordinal states. This can be visualized as in Figure S1, and has the result that RSN-states that are nearer in ordinal label are considered nearer in the corresponding state space; for example RSN-state 2 can be considered nearer to RSN-states 1 or 3 than to RSN-state 12.

We derive this distance metric from the transition matrix directly. Let Θ be the Markov state transition matrix, such that each entry $\theta_{i,j} = P(z_{t+1} = j \mid z_t = i)$; that is, each entry contains the probability of observing a direct transition from state *i* into state *j*. First, we exclude self-transitions, which are uninformative for the purposes of distances between states; this is done by setting the diagonal entries to zero, then normalizing so each row sums to one, such that our new matrix has entries $\psi_{ij} = P(z_{t+1} = j \mid z_t = i, z_{t+1} \neq i)$. Noting that high transition probabilities should correspond to small distances, we then define the distance between two states as the probability of not observing that state transition:

$$d_{ij} = 1 - \psi_{i,j}$$

Finally, to convert this to a symmetrical matrix of distances (as currently $d_{ij} \neq d_{ji}$), we simply average corresponding off-diagonal entries $\widehat{d}_{ij} = \frac{1}{2}(d_{ij} + d_{ji})$.

Given the 12 × 12 matrix of distances $\widehat{d}_{ij}$, we then use multidimensional scaling to identify the single axis that accounts for the most variance in the distance matrix. RSN-states are then labeled 1-12 in the order of their appearance on this axis – see visualization in Figure S1.

RSN Spectral Information

Each RSN-state is defined by a distinct spatial distribution of spectral power and coherence. These characteristics can be interpreted directly from the observation model parameters - however this may emphasize certain traits of the model fitting procedure, in particular the effects of the PCA dimensionality reduction methods applied, rather than the underlying spectra that is observable in each RSN-state. For this reason, we extract the spectral information by fitting a multitaper to the raw data, conditioned on the active RSN-state as introduced by Vidaurre et al. (2016) which provides an empirical assessment of the power and coherence for each ROI as a function of frequency. We fit the multitaper using the following parameters: a taper window length of 2 s; frequency resolution of 0.5Hz; frequency range of 1 to 45 Hz; and applying 7 Slepian tapers. As outlined above, due to the increased confidence of spatial RSN-state parameters derived from dataset C, a larger dataset for which MRI structural information was available, the spatial and spectral distributions per RSN-state referred to in this paper were derived from this dataset, supporting their interpretation as a set of canonical RSN-states.

This information is still very high dimensional so we summarize it through a spectral mode decomposition, obtaining spatial power and coherence maps for a data-driven set of frequency band modes. This decomposition is implemented by non-negative matrix factorization as detailed in Quinn et al. (2018) and Vidaurre et al. (2018). For summary purposes in Figure 2B and Figure S1, we fit this with two modes, to separate a single wideband mode from higher frequency noise. To explore further the spatial breakdown of different frequency modes, we fit the same non-negative matrix factorization procedure with four modes as in Figure 5 (use of four modes was found to produce broadly stable decompositions and mirrors findings in previous work). These can be derived for each RSN-state; alternatively, to more accurately reflect the full power and coherence distribution at a particular event time, we can use the evoked RSN-state distribution to weight the power and coherence information, producing HMM regularized time frequency plots in Figure 5 and Figure S4.

Replay Evoked RSN Analysis

Figure 2A analyses the average RSN-state distribution evoked by individual replay events. At the single subject level, we took the probabilistic RSN-state time course $\{\gamma_{i,t}\} = P(Z_t = i)$; that is, a timeseries with K distinct values between 0 and 1 at each timestep reflecting the probability of each RSN-state's activation. We then baseline corrected by subtracting the average over all time points for that subject, such that values greater than zero reflect probabilities greater than the session average, and values less than zero reflect activation probabilities below the session average for that subject. We then epoched this time series using the replay times identified above, extracting a window extending from 0.5 s prior to each event to 0.5 s after each event. For each subject, we then computed the evoked RSN-state distribution over this time window by averaging the epoched RSN-state distributions over all replay events. Let us denote by $B_{i,\tau,n}$ the evoked distribution for subject *n* and RSN-state *i* at timelag τ from the estimated replay events. Figure 2A plots the mean of these values over subjects, reflecting the expected increase or decrease in RSN-state probability evoked by individual replay events.

To test the significance of these results two types of statistical test were utilized; to support claims of a significant result at a specific point in time we used a one-sided t test, whereas to support claims of a broad peak of significant points across time we used a non-parametric sign flipping cluster permutation test. The cluster permutation test determined the probability of finding by chance a cluster of consecutive significant points that was the same size or larger than that observed in the data. We defined significance in this context as observing a group level t-statistic exceeding 3; we then generated a null distribution of cluster sizes by randomly permuting 5000 times the sign of each subject's evoked response parameters $B_{i,\tau,n}$ before computing group t-statistics, and determining the number of consecutive points exceeding the significance threshold. We could thus compute the chance probability of generating a cluster of the same size observed in the data.

When testing whether this result replicated on a second study, we applied an inflexible analysis to determine cluster significance. Specifically, we took the clusters in time found in the analysis of Dataset A. We then extracted the corresponding data for that time cluster in Dataset B and defined our cluster threshold as the minimum t-statistic within this temporal cluster. In a final step we then

computed (using the same sign-flipping permutation tests) the probability of a cluster of points the same or greater in size than this threshold occurring by chance in Dataset B.

Figure 2C plots the results of the same analysis on the functional localizer data; epochs were defined by the time of visual stimulus presentation, corresponding to t = 0 in the plot. The thick red line indicates the time at which the replay classifiers were trained.

Figure 2D then directly compares the evoked RSN-state distribution between the replay and functional localizer data sessions; for ease of presentation, we only focused on the distribution at the exact time of replay onset (t = 0) and the exact time point the replay classifiers were trained on (t = 200msec); for each of the 12 RSN-states we conducted a paired t test to check whether the evoked RSN-state distributions were significantly different across the two conditions.

Controlling for Replay Classifier Variance During DMN Activation

RSN-states 1 and 2, which correlate with replay, also have the highest variance in the data (as in Figure 4, these RSN states have the highest broadband power and therefore the highest variance). This leads to increased variance in the classifier outputs when these states are on, which could lead to spurious "reactivations" occurring solely as a result of the increased variance in the classifier outputs.

To control for this, we note that such spurious effects must be unbiased; that is they should not selectively bias a classifier output toward a particular value. It therefore follows that, when classifier outputs are plotted by percentile, any spurious effects attributed to increased variance should be symmetric across the median. Thus, we repeated the replay evoked RSN analysis while varying the threshold value from 1% to 99% (see Figure S6). This allowed us to characterize the degree to which the relationship was dependent upon the choice of threshold value, and also whether the effect was symmetric over thresholds (suggesting it is driven by the variance of classifier outputs) or strongly weighted toward higher threshold values (suggesting it is driven by the classifier values). These are shown in Figure S6.

Comparing Reactivation Evoked and Replay Evoked Distributions

Our main result focuses on the replay evoked distributions; in Figures S6F and S6G we compare this with the reactivation evoked distribution to relate our findings to the extensive literature on reactivations. This analysis was performed by repeating the precise steps outlined above for Replay Evoked RSN Analysis, but replacing the replay time courses with the reactivation time courses – that is, the actual stimulus classifier outputs denoted by $A_t, B_t, \ldots$ in the Replay Detection methods outline.

These two measures are however strongly correlated (Pearson's Rho = 0.45), as we have defined the replay time course itself as a linear superposition of the reactivation time courses. Furthermore the autocorrelation structure of the reactivation time courses and the bursty nature of the replay time course characterized below impose fundamental methodological constraints this specific analysis, such that Figures S6E and S6F should be interpreted accordingly.

Spectral Characterizations of Replay and Classifier Outputs

A growing field of literature focuses on the dynamics of classifier outputs using spectral analysis methods (Kerrén et al., 2018). Such studies may, for example, use some cued paradigm, training multiple classifiers on each successive time point after stimulus onset and then analyze in detail the Fourier spectrum of the cross validated accuracy time course over trials. Readers familiar with such analyses may be interested in how they would apply to our paradigm.

Our methods are different in a number of important ways that limit the potential of these common analysis techniques when applied to our data. First, these analyses rely upon the use of aligned trial data, allowing independent classifiers to be applied to the data at each consecutive timestep from the stimulus presentation time. In contrast, we take a single classifier for each stimulus (taken from the point of maximum decoding accuracy, 200msec following stimulus onset in the functional localizer data) and apply that classifier to the entire timeseries of resting state data. Consequently, whereas the approach as applied by Kerrén et al. (2018) typically identifies complex spectral characteristics emerging in the decoded signal, in our analysis the spectra of the classifier outputs is by definition a static linear function of the cross spectrum (because the decoding weights being used are fixed over time).

Nonetheless, this can still be informative, and when we apply this analysis to our data in Figure S3E we find the spectrum closely mirrors that of the RSN-states that align to replay; specifically, it reproduces two prominent peaks in the alpha and delta/theta range. These peaks are accentuated when we time-lock this analysis to epochs surrounding replay events; extracting the same modes in this data as used in the main manuscript text, we find a prominent increase in the alpha range (paired t test, p = 0.002) and a weaker but significant increase in the delta/theta range (paired t test, p = 0.045). Due to the methodological differences with the analyses of Kerrén et al. (2018), as highlighted above, the interpretation of these peaks is more limited. These oscillatory patterns are stronger at replay times, but we do not know whether these reactivations are themselves a function of these oscillations or merely coincident with them.

Some readers may be interested in what such an analysis would conclude when applied to the replay time courses. However, such an analysis would be inappropriate in this case. Spectral analysis applies well to data that has a roughly Gaussian distribution; it is typically applied to the 'classifier evidence', namely the classifier output before a sigmoid nonlinearity is applied. However, as the replay time course is determined by a logic operation (see section on Replay Detection), there is no equivalent to the 'classifier evidence' time course upon which these analyses are conducted; the replay time course itself is highly nonlinear as shown in Figures S3B and S3C. The shape of this time course led us to conclude that it was more appropriately modeled as a Poisson point process,

CellPress
OPEN ACCESS

whose dynamics are better represented by statistics such as the Fano Factor, than by a spectral analysis such as pursued by Kerrén et al. (2018), and by others in similar papers.

Transient Burst Activity Analysis

To analyze the temporal statistics of replay onset, we modeled the estimated replay event times for each subject and as a Poisson point process. Wherever an interval between events encompassed MEG data samples that had been marked as bad, the whole interval was excluded. We then partitioned the replay time course into non-overlapping windows, for a given window length W, and computed the total sum of replay events observed in each window (Teich et al., 1996). The Fano Factor, for a given window of length W, is then given by:

$$F_W = \frac{\sigma_W^2}{\mu_W}$$

Where μ_W and σ_W^2 are the mean and variance of event counts in each window. The Fano Factor was computed individually for each subject, Figure 3D plots the mean ± ste over subjects for each window length. We then computed parametric t tests on the Fano factor to test the null hypothesis that it was equal to one.

This alone does not prove conclusively that replay occurrences are transient burst events, but rather that they are not homogeneous Poisson processes. Hence, we furthermore tested a much broader null hypothesis that the intervals between replay events were independent and identically distributed, following the approach of Teich et al. (1996). We generated surrogate data by shuffling the interval times; that is, for each subject, we took the replay event time course, computed the intervals between consecutive events, and randomly permuted the intervals to generate a new event time course. This maintained the exact distribution of replay intervals while removing any dependence between consecutive intervals. We then computed the subject and group Fano Factors exactly as above. With this surrogate data, after 1000 permutations the maximum Fano Factor computed (plotted as the dotted line in Figure 3D) remained well below that of the true time course, confirming that the longer term dispersion arises from correlation over consecutive intervals ($p < 1e\text{-}3$) and allowing us to conclude that replay is characterized by irregular transient bursting behavior.

We then repeated the analysis for RSN-state activations (Figure 3E). Treating each RSN-state visit as an event, we removed consecutive activations and just used the interval between events to create the equivalent RSN-state visit event time course. As above, we computed the Fano Factor versus window length for each subject.

To compute the significance of these results, we used a one-way ANOVA to test the null hypothesis that there was no significant difference between the Fano factors of different RSN-states; this was conducted for each window length resulting in 100 multiple ANOVA tests. We only report the highest p value obtained, which remained significant with Bonferroni correction, allowing us to reject the null hypothesis for all window lengths observed. We could then assess a two-sample t test, taking each RSN-state one by one and testing the hypothesis that the observed Fano Factors for that RSN-state were no different from the entire population of remaining RSN-states. For example, we would take the 21 subject level Fano Factors for RSN-state 1, and compare that with the population of size 21 by 11 of Fano Factors for each subject for RSN-states 2-12, then repeat this for RSN-state 2. Again, this test was computed for each value of the window size; only the highest obtained p value was reported, all others were below this and therefore more significant.

Finally, Figure 3F considers the interval between replay events given the active RSN-state; that is, if a replay event occurs at time t we compute the length of time to the successive replay event, and also the most likely RSN-state to be active at time t. In this analysis, we omit any observations where the interval to the next replay event overlaps with segments marked as bad. For each subject, we thus compute a mean replay interval given the active RSN-state, generating a collection of 21 observations of replay intervals given the active RSN-state. Certain subjects had very few observed replay events in particular RSN-states, resulting in high variance estimates; we therefore omitted from further analysis any means that were derived from fewer than ten observations. We then could then test for significant variation by active RSN-state with a one-way ANOVA followed by two sample t tests, in which we tested whether the replay intervals given RSN-state k was active were significantly different from the replay intervals of all other RSN-states combined.

RSN-state Power and Coherence Analysis

Figure S4 is another way to characterize the spectral information unique to each RSN-state. For these plots, we take the RSN-state spectral information as outlined by the multitaper approach outlined above.

To summarize the wideband response over all frequencies, we take the NNMF decomposition with two modes – that is, with one mode that we interpret as higher frequency noise, and the remaining wideband mode interpreted as a denoised average of the overall frequency spectrum – and plot the RSN-state specific power and coherence for each ROI, given the active RSN-state. The power is given directly by the multitaper approach; coherence is defined per pair of ROIs, so the values plotted on the y axis in these plots is the sum of coherences between a given ROI and all others.

We can also follow the same procedure for each of the three spectral decompositions corresponding to individual spectral modes; that is, we derive the power and coherence for each ROI and for each given RSN-state, as computed for each of the delta / theta, alpha and beta spectral modes.

Replay Evoked Spatial Maps

To accurately convey the spectral profile that is linked to each replay event, we can combine the replay evoked RSN-state distribution plotted in Figure 2A with each RSN-state's spectral information. As outlined above, we have values for the spectral power and coherence of each RSN-state from dataset C. We can initially visualize these by averaging over all recorded ROIs; the left-hand side of Figures 5A and 5B plot the state specific power and coherence values, respectively. To compute the power and coherence patterns that most closely reflect replay times, we take the subject specific replay evoked RSN-state distributions, referred to as $B_{i,\tau,n}$ above and plotted in the lower panel of Figures 5A and 5B. For consistency of interpretation with Figure 2 these are plotted after baseline correction, however to compute the replay evoked power and spectral density plots we revert to the non-baseline corrected values, such that each $B_{i,\tau,n}$ is a probability between 0 and 1, with the sum of values over all different states equal to 1 for each time point and subject. We can then compute the expected power and coherence as a function of both time and frequency around each replay event by weighting the state specific PSD and coherence values by the replay evoked state distribution. Note that this analysis was done at the subject level; that is, we computed subject-specific power and coherence estimates as a function of both time and frequency using each subject's specific replay evoked state distribution. This produced maps of dimension [time x frequency x subjects x channels] in the case of PSD and [time x frequency x subjects x channels x channels] in the case of coherence. Given the high dimensionality of this data we separately summarize the time-frequency information (Figures 5A and 5B) and the spatial frequency distribution (Figure 5C). For visualization in the heatmaps of Figures 5A and 5B we baseline corrected at the subject level, such that zero on the color scale denotes an average PSD and coherence. Figure 5C then expresses how this information is distributed over frequency and space. This plot uses only the distribution defined at t = 0, the exact time of replay estimated by Liu et al. (2019). We decompose the spectral information using the previously introduced non-negative matrix factorization, a data-driven approach that identifies the main spectral modes explaining the data, producing a vector of power values over channels and a matrix of coherence values between channels for each spectral mode and each subject. For the purposes of visualization, PSD values are thresholded by the highest 90% of values. The higher dimensionality of the coherence matrix required a more stringent thresholding approach; for this we applied a Gaussian mixture model threshold test (Quinn et al., 2018; Vidaurre et al., 2018), only plotting connections if they fall into a distinct mixture separated from the global distribution. All these spectral estimation and thresholding steps follow those previously introduced (Quinn et al., 2018; Vidaurre et al., 2018).

High Frequency Oscillations

To analyze the higher frequency correlates of specific RSN-state activations, we first took the timeseries of state activations that had been fit to the resting state data as outlined above. Importantly, this analysis had been conducted on data that was downsampled to 250 samples/second and band pass filtered between 1 and 45Hz. For this analysis we therefore returned to the raw data which had been acquired at 600samples/second with a low pass filter applied with cutoff 160Hz. This data did contain power line noise with a baseline frequency at 50Hz and some harmonic noise at integer multiples of 50Hz; we did not attempt to filter this out to avoid introducing any undesirable filter effects. Using the exact state activation times and multitaper approach outlined above, but instead using data with a higher sampling rate that included high frequency content, we estimated the power spectral density associated with each state over all frequencies from 1 to 160Hz. Figure 6A plots the mean ± standard error over subjects of the PSD across this broader frequency band for all states. To compare this to the PSD estimated at replay times, we reused the same multitaper approach but replaced the state activation times with estimated replay times. Specifically, we defined a window of width 30msec around the time of each estimated replay event, and computed the PSD across all frequencies for the data in these windows using the same multitaper approach. To estimate the baseline non-replay high frequency power, we followed the same approach but now using randomly sampled replay times that were shuffled over subjects – this ensured consistency in epoch lengths and temporal profiles while extracting data that was not aligned to replay events in any consistent way. These PSD values are plotted in Figure 6B. Finally, given the exclusive correlation between high frequency power increases and RSN-state 2, we explored the spatial distribution of power in higher frequencies in this specific RSN-state. Figure 6C averages the PSD values over all subjects between 102-148Hz (this band was defined to exclude mainline power noise effects) for each ROI, and plots the spatial topography over all ROIs.

Baseline Correction for High Frequency Oscillations

The replay evoked spectrum in higher frequencies in Figure 6B may appear more broadband than previously reported in Liu et al. (2019). This is in fact due to the baseline correction measures used, with a more narrowband signal emerging when the signal is plotted relative to baseline than when the absolute signal is plotted.

To compare our findings directly, we recreated Figure 6B using a wavelet decomposition applied to the entire timeseries. This allowed us to compare our replay evoked distribution of Figure 6B – that averages over a time window around replay events – with the full epoch analysis reported by Liu et al. (2019) which plots the replay evoked spectrum as a function of both time and frequency around replay events. To produce Figure S5D we took the same 30msec window around replay events which replicates the broadband high frequency spectrum of Figure 6B. We then plotted the difference between this signal and baseline as in Figure S5E. Finally, we plotted this spectrum as a function of both time and frequency in Figure S5F, replicating the figures reported by Liu et al. (2019).

Neuron, Volume *109*

Supplemental information

Replay bursts in humans coincide with activation of the default mode and parietal alpha networks

Cameron Higgins, Yunzhe Liu, Diego Vidaurre, Zeb Kurth-Nelson, Ray Dolan, Timothy Behrens, and Mark Woolrich

Supplementary Information

This PDF file includes:

Figs. S1 to S6

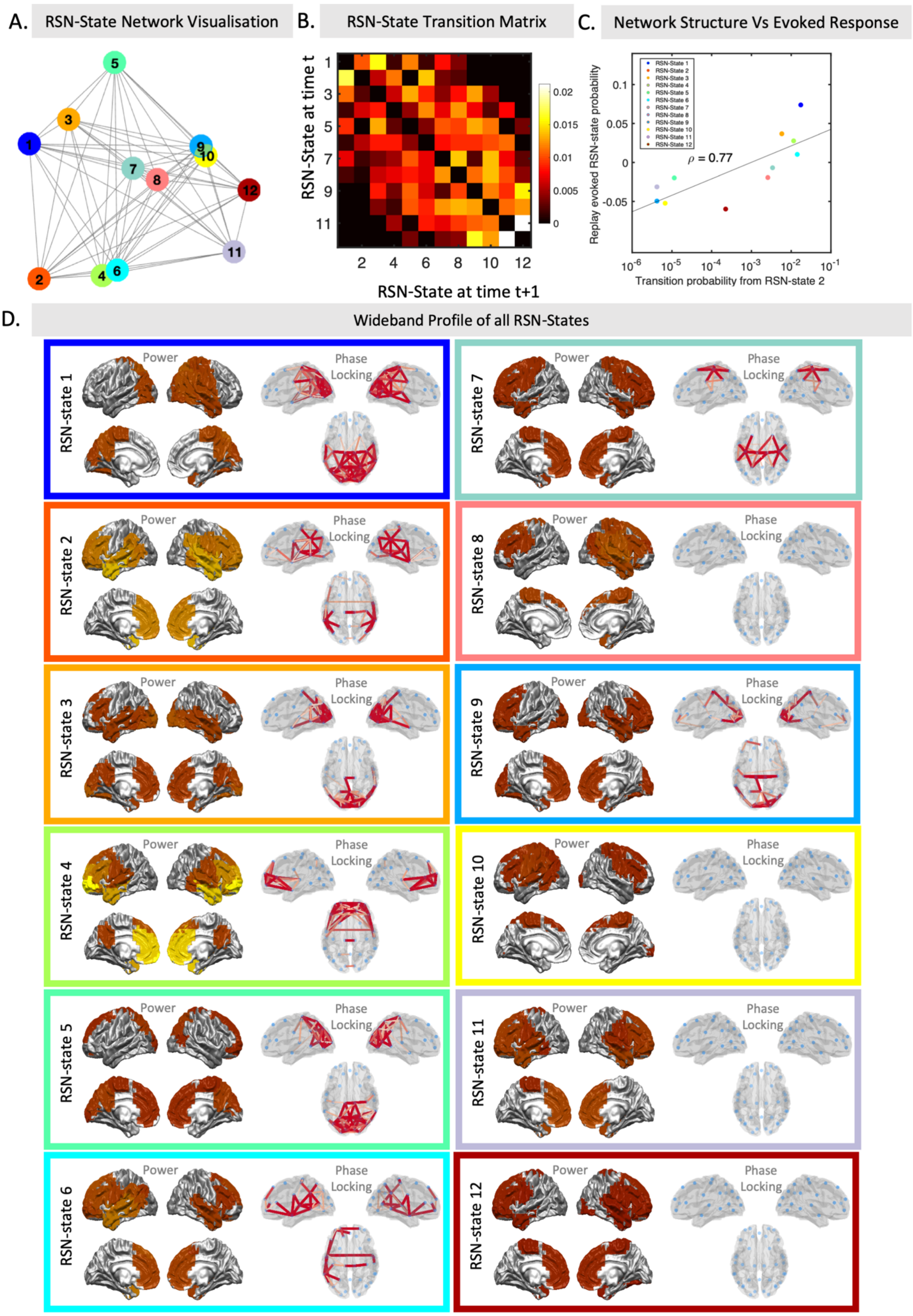

A. RSN-State Network Visualisation
B. RSN-State Transition Matrix
RSN-State at time t
RSN-State at time t+1
C. Network Structure Vs Evoked Response
Replay evoked RSN-state probability
Transition probability from RSN-state 2
ρ = 0.77
D. Wideband Profile of all RSN-States
RSN-state 1
RSN-state 2
RSN-state 3
RSN-state 4
RSN-state 5
RSN-state 6
RSN-state 7
RSN-state 8
RSN-state 9
RSN-state 10
RSN-state 11
RSN-state 12
Power
Phase Locking

Fig. S1.

Resting State Network Profiles; related to Figure 2. (A) The RSN-states can be visualized as a full network through a multidimensional scaling of the transition matrix (see STAR Methods). We label RSN-states 1-12 in order of their appearance on the horizontal axis of this graph, accounting for the greatest portion of distances between RSN-states. (B) The transition matrix (excluding diagonal entries) for the RSN-states; the diagonal structure reflects this labelling structure, with transitions more probable between adjacent pairs of RSN-states than distant pairs. (C) Correlating the second row of the RSN-state transition matrix on the x axis with the replay evoked RSN-state probability (ie the value at t=0 on Fig. 2A) on the y axis identifies a strong relationship (Pearson's rho=0.77, p=5.1e-3). (D) Wideband profiles for all RSN-states, plotting the wideband power (thresholded at 50%) and coherence (thresholded with Gaussian Mixture Model). See STAR Methods for further details and Supplementary Text for clarification of how these relate to equivalent networks in fMRI.

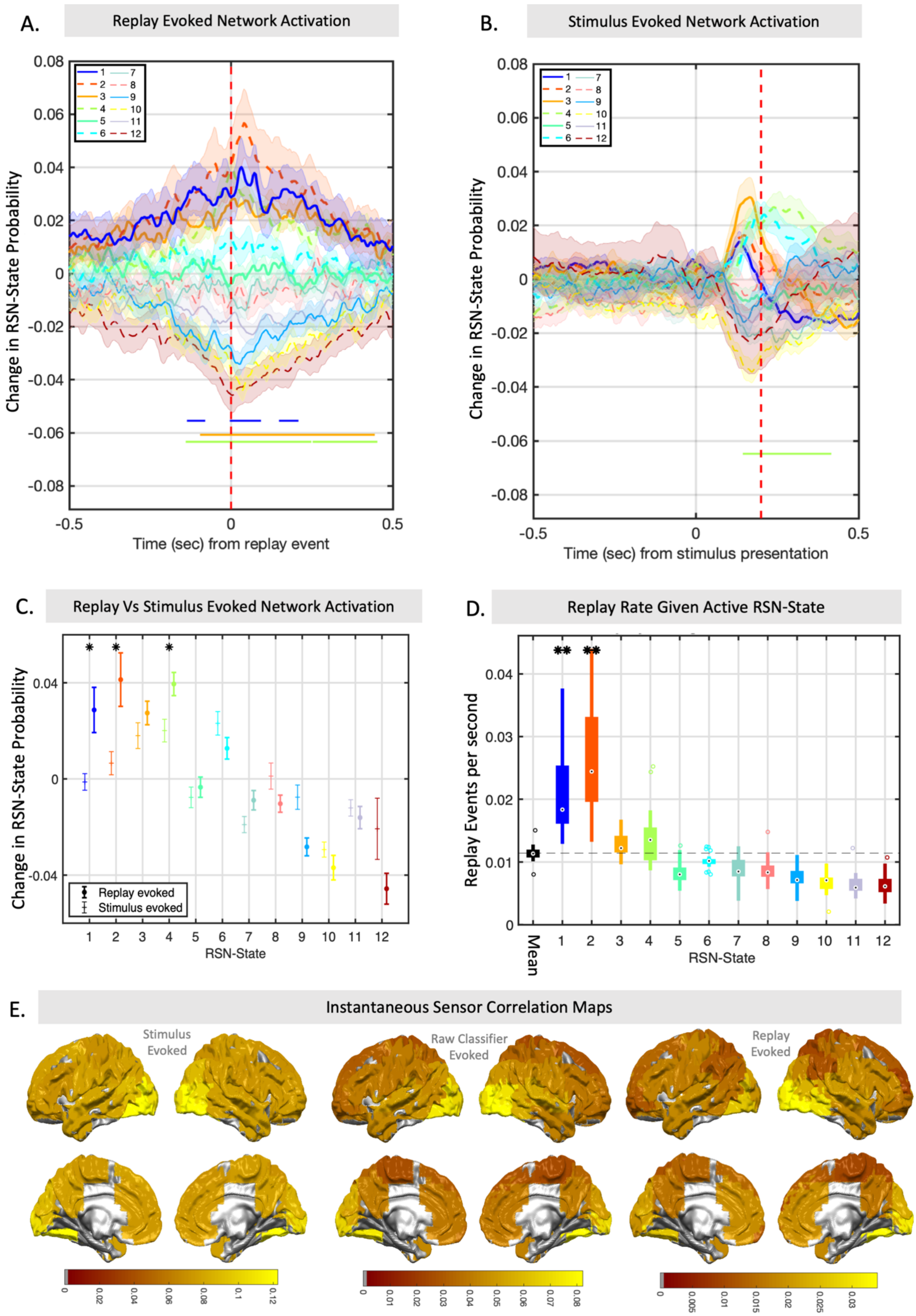

A.
Replay Evoked Network Activation
Change in RSN-State Probability
Time (sec) from replay event
B.
Stimulus Evoked Network Activation
Change in RSN-State Probability
Time (sec) from stimulus presentation
C.
Replay Vs Stimulus Evoked Network Activation
Change in RSN-State Probability
Replay evoked
Stimulus evoked
RSN-State
D.
Replay Rate Given Active RSN-State
Replay Events per second
Mean
RSN-State
E.
Instantaneous Sensor Correlation Maps
Stimulus Evoked
Raw Classifier Evoked
Replay Evoked

Fig. S2.

Replicating the result of Figure 2 on a second dataset and further justification of results. (A) Replication of Fig. 2A on a second dataset; plotting mean +/- ste of the RSN-state probability evoked by replay onset; replay onset coincides with a broad peak in activity in RSN-states 1 and 2, and to a lesser extent RSN-states 3 and 4. Significance bars show clusters where $p<1e-4$. (B) Replication of Fig. 2C on a second dataset; mean +/- ste of the RSN-state probability associated with the original stimulus data. Significance bars show clusters where $p<1e-4$. (C) Replication of Fig. 2D on a second dataset; comparing directly the mean +/- ste of the evoked state distribution at replay time and at the classifier training time identifies RSN-states 1 and 2 as significantly increased during spontaneous replay (multiple paired t tests). Single asterisk denotes $p<0.05$, double asterisk denotes $p<1e-3$. (D) Replay rate as a function of active RSN-state; in Fig. 2 we showed that replay onset predicts an increase in RSN-states 1 and 2. Reinforcing this finding, here we analyze this relation in reverse. We show that activation of RSN-states 1 and 2 similarly predict an increase in the rate of replay events. Asterisk denotes significance at $p=0.05$, double asterisk denotes significance at $p=1e-3$. (E) Instantaneous sensor correlation maps; putting aside the network patterns analyzed in the main text, we can similarly consider the instantaneous sensor magnitudes and how they correlate with (i) the design matrix entries in the original stimulus encoding data (left); (ii) the raw classifier scores in the resting state data (middle); or (iii) the replay scores in the resting state data. This analysis confirms the underlying evidence behind each of these signals are representations predominantly in visual cortical areas.

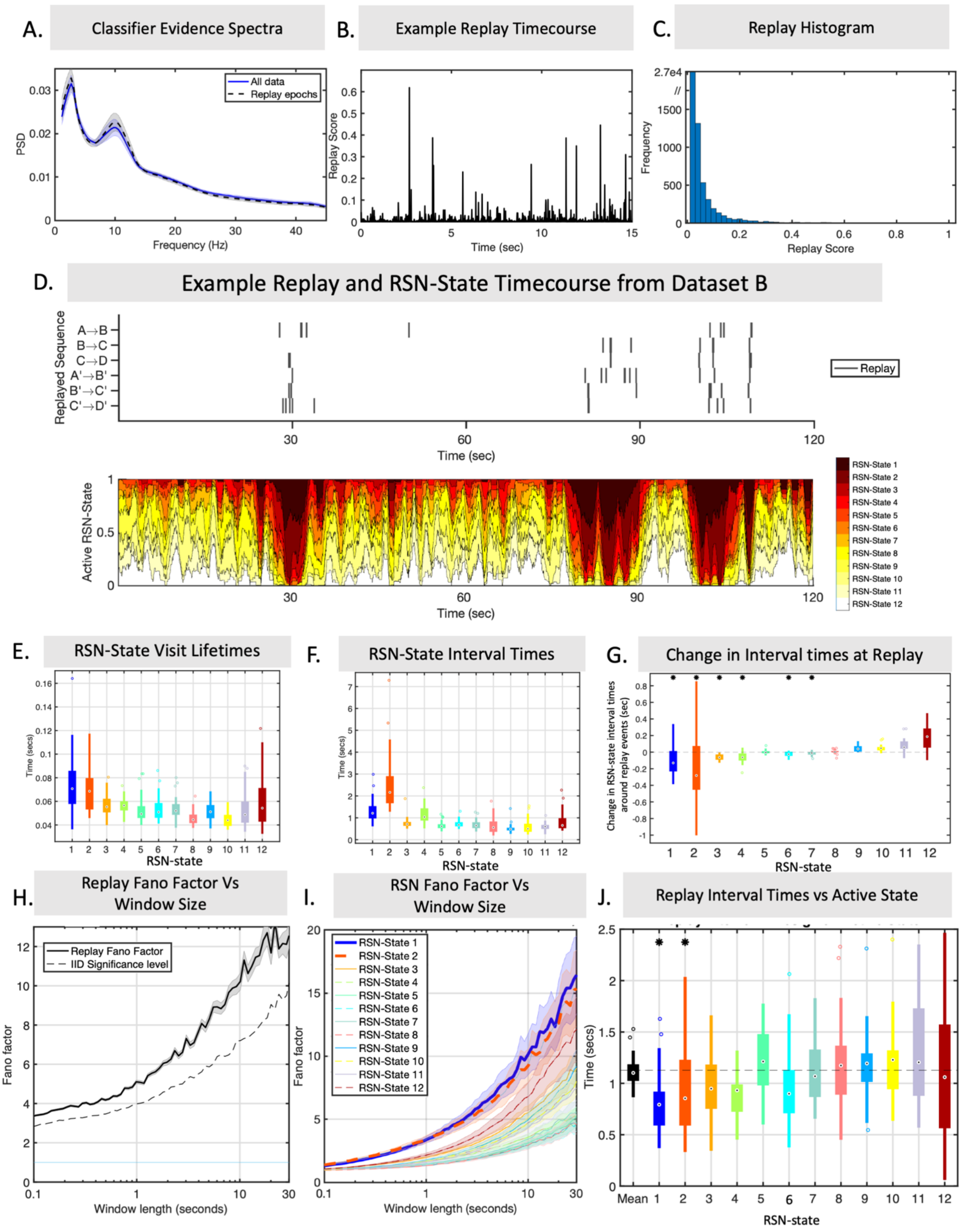

Fig. S3.

Replicating the results of Figure 3 on a second dataset and characterizing the replay timecourse. (A) The classifier evidence spectra reproduces peaks in alpha and delta/theta modes characterized in the text. The two lines plot the power spectrum of the classifier timecourses before the logistic sigmoid transform is applied; this is separated by replay epochs and non-replay epochs as a baseline. This analysis reproduces two prominent peaks in the alpha and delta/theta range broadly characterized in the data, showing that neither of these

features are removed by the classifiers. These peaks are accentuated when we time-lock this analysis to epochs surrounding replay events; extracting the same spectral modes in this data as used in the main manuscript text (see STAR Methods), we find a prominent increase in the alpha range (paired t-test, $p=0.002$) and a weaker but significant increase in the delta/theta range (paired t-test, $p=0.045$). (B) Example replay timecourse from one subject. (C) Histogram of replay timecourse values; this timecourse is very sparse, with the vast majority of values very close to zero. This justifies the binarized dynamics analysis pursued in the text over alternative spectral analysis methods. (D). Replication of Fig 3A on second dataset; example data from one subject of the replay event times (upper) and one-second moving average RSN-state probabilities (lower), suggesting both replay and RSN-state visits are not distributed evenly over long timescales. (E) Group level RSN-state visit lifetimes, characterizing the variation in mean lifetime statistics over subjects. (F) Group level RSN-state interval times, characterizing the variation in mean interval time statistics over subjects. (G) Change in interval times at replay; the mean RSN-state interval times at replay (defined as a state occurrence within 250msec of a replay event) minus the mean RSN-state interval times outside replay periods. This identifies a number of RSN-states, most prominently RSN-states 1 and 2, as having shorter interval times around replay events, confirming these state visits cluster together around replay events. Asterisk denotes significance with left-tailed paired t-test at $p<0.05$. (H) Replication of Fig. 3C on a second dataset; temporal irregularity can be quantified by looking at the Fano factor as a function of window size. Replay events show that this irregularity measure increases over longer timescales, displaying maximum temporal irregularity over windows of ten seconds or more. (I) Replication of Fig. 3D on a second dataset; this structure is replicated by the RSN-state activations, with RSN-states 1 and 2 displaying the most irregular patterns at long timescales. (J) Replication of Fig. 3E; this temporal structure is not just common but in fact coincides; replay events that occur during RSN-state 1 or 2 have significantly shorter intervals, reflecting rapid bursty behavior during the infrequent state visits and long periods of quiescence outside of these. Asterisk denotes significant deviation from mean at $p<0.05$.

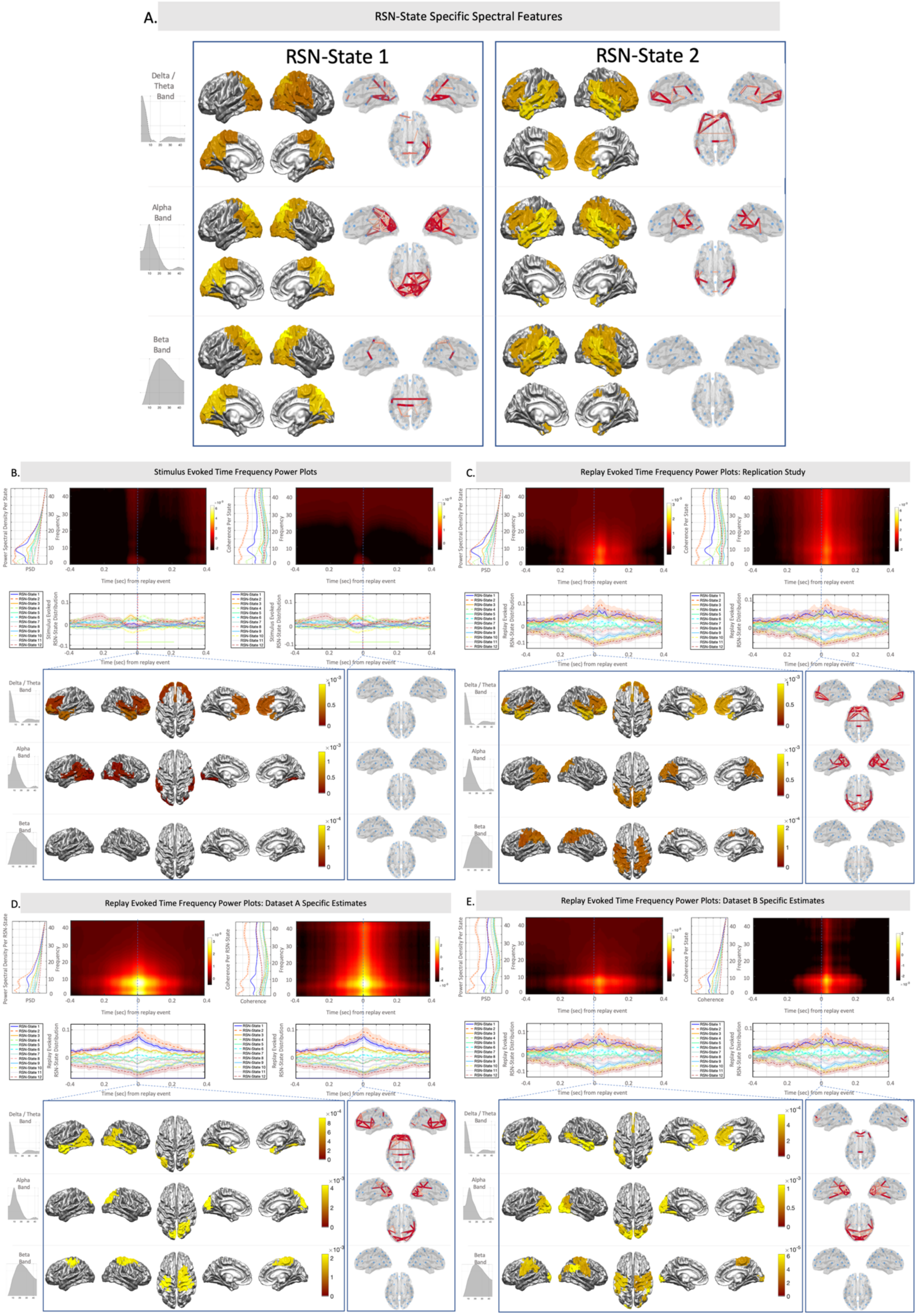
A.
RSN-State Specific Spectral Features
RSN-State 1
RSN-State 2
Delta / Theta Band
Alpha Band
Beta Band
B.
Stimulus Evoked Time Frequency Power Plots
C.
Replay Evoked Time Frequency Power Plots: Replication Study
D.
Replay Evoked Time Frequency Power Plots: Dataset A Specific Estimates
E.
Replay Evoked Time Frequency Power Plots: Dataset B Specific Estimates
Time (sec) from replay event

Fig. S4.

Replication of Figure 5 results on a second dataset and further spectral profile characterizations. (A) The full spectro-spatial activity maps associated with RSN-states 1 and 2; where Fig. S1 plotted the wideband distribution of power, this can be further analyzed using a data driven spectral decomposition. We here plot the spatial distribution of power in each frequency band (thresholded at 50%); along with the coherence network (Gaussian mixture model threshold; see STAR Methods). This supports our interpretation of RSN-state 1 as the parietal alpha network, given the prominence of the alpha peak over parietal areas; this pattern of electrophysiology activity has been shown in simultaneous EEG/fMRI experiments to anticorrelate with the fMRI Dorsal Attention Network (Mantini et al., 2007; Sitnikova et al., 2020), supporting the interpretation that it is associated with the DAN 'switching off'. These spectral maps further support our interpretation of RSN-state 2 as reflecting the MEG DMN equivalent; further to the broadband patterns of activity in parietal cingulate and lateral parietal areas of Fig S1, the narrowband signal identifies frontal, anterior cingulate and lateral temporal areas active in a lower frequency band to the dominant alpha band. This is consistent with the previous MEG literature on the DMN (Vidaurre et al., 2018) and with simultaneous EEG and fMRI recordings of the EEG correlates of the fMRI DMN network (Mantini et al., 2007; Sitnikova et al., 2020). (B) As a control, we can apply the same analysis of Fig. 5 to the original stimulus encoding data. Using the original stimulus-evoked RSN-state probabilities as weights for the spectral information unique to each RSN-state allows a reconstructed time-frequency estimate of power spectral density and coherence around stimulus presentation. This does not replicate the same level of power and coherence seen around replay events, confirming these patterns are unique to spontaneous replay. (C) Replication of Fig. 5 using a second dataset; using the replay-evoked RSN-state probabilities as weights for the spectral information unique to each RSN-state allows a reconstructed time-frequency estimate of power spectral density and coherence around replay events, revealing a prominent peak in the alpha and delta /theta bands. Plotting the spatial distribution of activity in the defined frequency modes at the time of replay identifies independent modes of coherent activity; a low frequency mode (top panel) comprising frontal DMN and temporal areas, and an alpha frequency mode (middle panel) comprising parietal DMN regions and visual cortex. (D) Replication of Fig. 5 using Dataset A spectral profiles; Fig. 3 was generated using RSN-state spectral information learned from Dataset C (see STAR Methods). The same plot can be reproduced using the spectral information from Dataset A itself, reproducing the same features of the main plot. (E) Replication of Fig. S4C using Dataset B spectral profiles; similarly Fig S4C was generated using RSN-state spectral information learned from Dataset C, but can be replicated using spectral information learned from Dataset B. For ease of visualization and comparison of the full spatial patterns, power spectra in B-E were thresholded at 75%; coherence networks were thresholded with a Gaussian mixture model (see STAR methods).

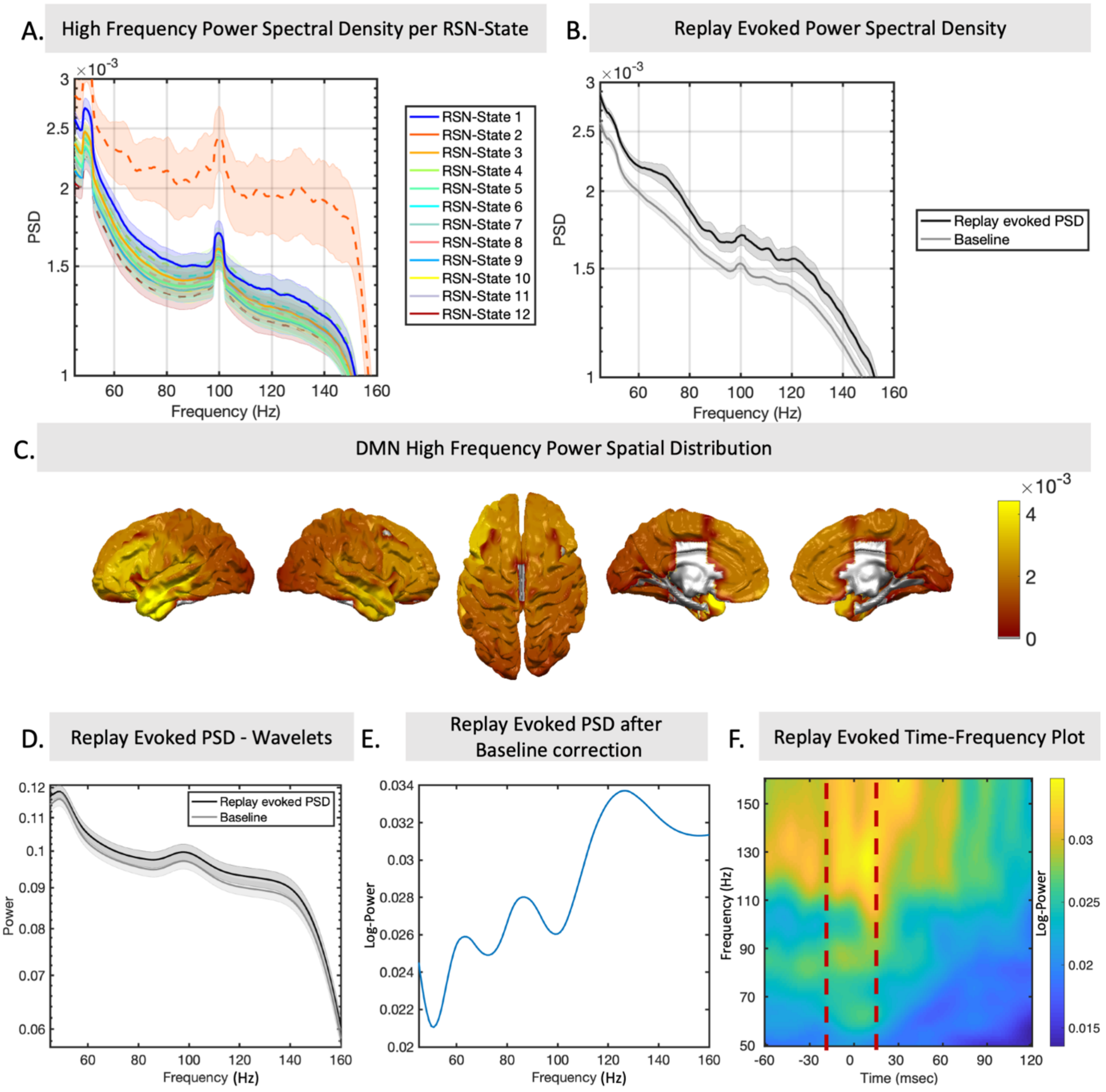

Fig. S5.

Replication of the result of Figure 6 on a second dataset. (A) Although the RSN-state model was originally fit to data filtered at 1-45Hz, we can still analyze whether the state timings correlate with specific patterns in frequency bands outside this range in the original data. This reveals a very strong association between the DMN state and power in high frequencies – despite these high frequencies not having been originally included in the model. (B) Similarly, the onset of replay is associated with an increase in high frequency power relative to the global average. Replay-evoked power computed by taking 50msec windows of data around identified replay events; for baseline see STAR Methods. (C) The spatial distribution of high frequency (102-148Hz) power in the DMN state; activity in this RSN-state and in this frequency band source localizes to temporal cortex. (D) To compare our findings directly with previously reported results (Liu et. al. 2019), we recreated Fig. 6B using a wavelet decomposition applied to the entire timeseries (see STAR Methods). Taking the same 30msec window around replay events replicates the broadband high frequency spectrum of Fig. 6B. (E) The replay evoked PSD after baseline correction; that is, the mean difference between a subject's replay-evoked log power and their log baseline power. This produces a stronger peak in higher frequencies than is apparent in D. (F) Plotting the full

time-frequency response at replay epochs after baseline correction reproduces the result reported by Liu et. al. 2019 that appears more narrowband than is apparent in D. Dotted red lines define the window that is used for ‘replay evoked power’ in D and E. A number of small differences remain in our preprocessing pipelines – specifically the use of beamformed and parcellated data as opposed to raw sensor signals, and the use of a 99% threshold as opposed to a 95% threshold when defining replay events – but the result reported by Liu et. al. 2019 is replicated here independently of those differences. Thus, we conclude that the relatively broadband appearance of the signal shown in Fig 6B appears more narrowband following baseline correction, which explains the apparent difference between this figure in our paper and the results previously reported by Liu et. al. 2019.

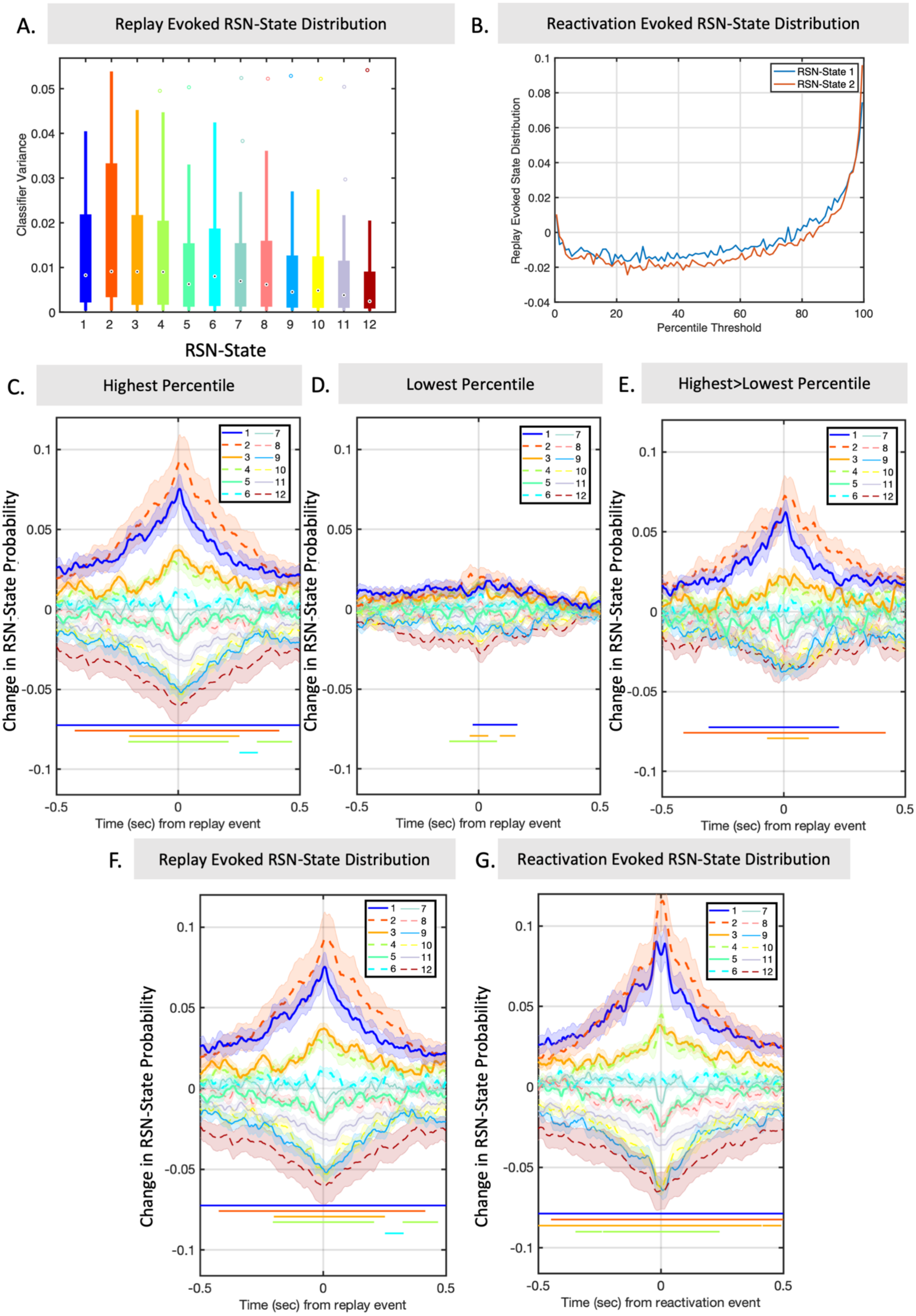
A.
Replay Evoked RSN-State Distribution
Classifier Variance
RSN-State
B.
Reactivation Evoked RSN-State Distribution
RSN-State 1
RSN-State 2
Replay Evoked State Distribution
Percentile Threshold
C.
Highest Percentile
D.
Lowest Percentile
E.
Highest>Lowest Percentile
Change in RSN-State Probability
Time (sec) from replay event
F.
Replay Evoked RSN-State Distribution
G.
Reactivation Evoked RSN-State Distribution
Time (sec) from reactivation event

Fig. S6.

Further controls and analyses; related to STAR Methods and Figure 2. (A) Variance of classifier output varies as a function of RSN-state (One way ANOVA, $p < 0.01$). Box plots show variation over subjects of observed classifier variance during activation of each RSN-state. (B) Focusing only on RSN-states 1 and 2, the replay evoked state distribution at $t=0$ (ie the exact time of estimated replay onset) is plotted against the percentile threshold used. This shows that the association with RSN-states 1 and 2 reported in Fig. 2A is robust over a broad range of percentile thresholds. It similarly performs the variance control outlined in the STAR Methods; this identifies a small effect emerging for the lowest 1 percentile, suggesting that the classifier variance does in some way contribute to the RSN-state replay relationship, however the effect is much weaker than the main result. (C) Replicating the main result of Fig. 2A, the RSN-state distribution evoked by the highest percentile of replay scores, for comparison. (D) The RSN-state distribution evoked by the lowest percentile of replay scores. This effect however is not significant after Bonferroni correction, and is only a fraction of the effect size of (D). (E) Taking the contrast of these as a control for the effect size that is solely due to classifier variance again replicates a strong association between RSN-states 1 and 2 and the onset of replay, confirming our interpretation of the main result. (F) The replay evoked RSN-state distribution is a special case of the reactivation evoked RSN-state distribution; the main result of Fig. 2A, reproduced for ease of comparison, with (G) the corresponding RSN-state distribution evoked by reactivation (see STAR Methods and Supplementary Text). Significance bars denote clusters with $p<1e-4$. We find no evidence of distinct relationships between RSN-states and reactivation as opposed to replay, however also caution against any strong interpretation of this result given the fundamental limitations of our methods to make such a direct comparison (see STAR methods).
